# Atorvastatin liposomes in a 3D-printed polymer film: a repurposing approach for local treatment of oral candidiasis

**DOI:** 10.1007/s13346-023-01353-4

**Published:** 2023-05-15

**Authors:** Eman M. Nour, Salma E. El-Habashy, Michael G. Shehat, Marwa M. Essawy, Riham M. El-Moslemany, Nawal M. Khalafallah

**Affiliations:** 1 Department of Pharmaceutics, Faculty of Pharmacy, Alexandria University, 1 Khartoum Square, P.O. Box 21521, Azarita, Alexandria Egypt; 2 Department of Microbiology and Immunology, Faculty of Pharmacy, Alexandria University, Alexandria, Egypt; 3 Department of Oral Pathology, Faculty of Dentistry, Alexandria University, Alexandria, Egypt; 4 Center of Excellence for Research in Regenerative Medicine and Applications (CERRMA), Faculty of Medicine, Alexandria University, Alexandria, Egypt

**Keywords:** Antifungal resistance, Drug repurposing, Mucoadhesive dosage forms, Additive manufacturing, Buccal drug delivery, Oral thrush

## Abstract

Oral candidiasis (OC) is an opportunistic fungal infection, common amongst the elderly and the immunocompromised. Unfortunately, the therapeutic efficacy of common antifungals is imperiled by the rise of antifungal drug resistance. An alternative promising therapeutic option possibly contributing to antifungal therapy is drug repurposing. Herein, we aimed to employ novel pharmaceutical drug delivery for enhancing the emerging antifungal potential of the hypocholesterolemic drug atorvastatin (ATV). ATV-propylene-glycol-liposomes (ATV/PG-Lip) were prepared then integrated in 3D-printed (3DP) mucoadhesive films comprising chitosan, polyvinyl-alcohol and hydroxypropyl methylcellulose, as an innovative blend, for the management of OC. ATV/PG-Lip demonstrated good colloidal properties of particle size (223.3 ± 2.1 nm), PDI (0.12 ± 0.001) and zeta potential (-18.2 ± 0.3 mV) with high entrapment efficiency (81.15 ± 1.88%) and sustained drug release. Also, ATV/PG-Lip showed acceptable three-month colloidal stability and in vitro cytocompatibility on human gingival fibroblasts. The developed 3DP-films exhibited controlled ATV release (79.4 ± 1.4% over 24 h), reasonable swelling and mucoadhesion (2388.4 ± 18.4 dyne/cm^2^). In vitro antifungal activity of ATV/PG-Lip was confirmed against fluconazole-resistant Candida albicans via minimum inhibitory concentration determination, time-dependent antifungal activity, agar diffusion and scanning electron microscopy. Further, ATV/PG-Lip@3DP-film exceeded ATV@3DP-film in amelioration of infection and associated inflammation in an in vivo oral candidiasis rabbit model. Accordingly, the results confirm the superiority of the fabricated ATV/PG-Lip@3DP-film for the management of oral candidiasis and tackling antifungal resistance.

**Graphical abstract:**

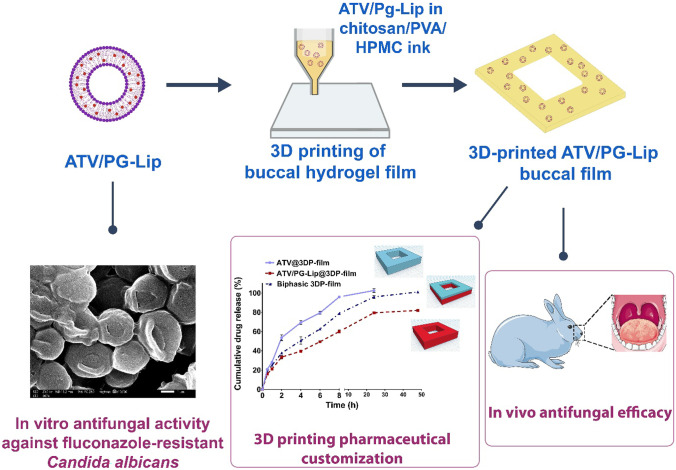

**Supplementary Information:**

The online version contains supplementary material available at 10.1007/s13346-023-01353-4.

## Introduction

Oral candidiasis is an opportunistic mucosal fungal infection that prevails among immunocompromised patients, including elderly, neonates, acquired immunodeficiency syndrome (AIDs) patients and patients receiving anticancer chemotherapeutics and immune suppressants [1]. In severe cases, infection may spread downward into the esophagus causing discomfort upon chewing, and thus impeding patients’ ability to swallow [2]. Among different candida species, *C. albicans* is responsible for 95% of oral candidiasis cases [3]. One of the most frequently prescribed antifungals for the treatment of oral candidiasis is fluconazole, a fungistatic triazole derivative [4]. Despite its effectiveness, the frequent use of fluconazole gave rise to acquired resistance [5]. *C. albicans* resistance to fluconazole reportedly results from the declined intracellular accumulation of the drug due to a decreased influx or enhanced efflux [5].

The upsurge of antifungal resistance triggered the need for more effective antimicrobial therapy [6]. However, the prolonged duration and the enormous cost of novel drug development pose challenges and shift the focus to antimicrobial drug repurposing as a promising therapeutic alternative, where drug safety has already been established [7]. Among drugs with promising antimicrobial repurposing potential is atorvastatin (ATV), a 3-hydroxy-3-methylglutaryl coenzyme A (HMG-CoA) reductase inhibitor that has a well-known plasma cholesterol-lowering effect. Its antifungal aptitude is described to be through suppressing the production of fungus cell wall ergosterol [8], yet the exact mechanism is still debatable. Recently, several studies reported the antifungal potential of ATV against *C. albicans* [8–10]. In one study [8], all tested candida strains were sensitive to ATV, while being resistant to fluconazole and nystatin. Also, Neto et al. demonstrated the in vivo efficacy of ATV emulgels against oral and vulvovaginal candidiasis [9]. Conversely, other results suggested the limited antifungal ATV activity against invasive candidiasis [10], probably due to ATV immune modulatory effect [11]. The controversy in literature associated with the repurposed antifungal potential of ATV still needs to be addressed. Accordingly, formulation of ATV in a nanodrug delivery system to enhance its repurposed antifungal efficacy could further be exploited.

Nanodrug delivery systems can improve the antimicrobial efficacy of repurposed drugs via improving solubility of poorly soluble drugs, enhancing permeability, decreasing effective dose and lowering dosing frequency, hence reducing overall toxicity [12]. A prominent example are propylene glycol-integrated liposomes (PG-Lip), which can efficiently serve as favorable deformable vesicular nanocarrier for drug loading and permeation through biological membranes [13]. The deformability of PG-Lip membranes allows them to squeeze through intercellular gaps of the cell membrane and transport their cargo into deeper tissue layers [14]. As reported by Kathuria et al., the skin permeability of tofacitinib citrate was enhanced by 4–11 folds after loading in propylene glycol liposomes [13]. To the best of our knowledge, PG-Lip aptness for buccal drug delivery was not previously investigated but is expected to similarly enhance permeation and efficacy of the delivered cargo.

The local management of oral candidiasis is generally preferred due to the ease of application and lower drug side effects compared to systemic delivery [12]. More specifically, employment of mucoadhesive films that bind to the oral mucosa can further extend the benefits of buccal delivery by controlling delivery of the loaded-drug, affording high patient comfort/compatibility and high dose accuracy compared to other dosage forms [15]. In this respect, the advent of three-dimensional (3D) printing as additive manufacturing technique has revolutionized the fabrication of tailorable drug delivery systems [16]. Specifically, extrusion-based 3D printing provides the feasibility for pharmaceutical customization of drug delivery system design and drug release pattern. For example, it was previously reported that the release profile of ibuprofen from polycarbonate-chitosan polymer blends varied with chitosan content [17]. Also, in our previous work [18], different doxycycline release patterns were obtained by developing two different biphasic designs (drug loaded-core and drug-loaded shell) using the same ink. Moreover, formulation of a nanodrug delivery system and further incorporation within a tailorable 3D-printed construct affected matrix porosity, swelling behavior and mechanical attributes [18, 19].

Given this basis, in this research, we attempted to investigate the formulation role of nanodrug delivery, via PG-Lip, as well as 3D printed mucoadhesive buccal film on ATV repurposed antifungal performance. To this end, the aim of this research was the development and characterization of ATV-loaded PG-Lip (ATV/PG-Lip), for the first time to the best of our knowledge. The developed ATV/PG-Lip were further formulated as innovative 3D-printed mucoadhesive buccal film (ATV/PG-Lip@3DP-film), comprising chitosan, polyvinyl alcohol (PVA) and hydroxypropyl methyl cellulose (HPMC) as novel biofunctional polymer blend. The developed composite 3D films were characterized for physicochemical features. Afterwards, an investigation was conducted to study the repurposed antifungal potential of the developed systems in-vitro and in vivo in a rabbit oral candidiasis model.

## Materials and methods

### Materials

Lipoid S100 (phosphatidylcholine; 95.8%) from soybean lecithin was a kind gift from Lipoid GmbH (Germany). Atorvastatin calcium (ATV) was a kind gift from Borg Pharmaceutical Industries (Egypt). Propylene glycol (PG) was obtained from alpha chemika (India). Hydroxypropyl methylcellulose (HPMC, K4M) and cholesterol were obtained from Sigma Aldrich chemical Co. (UK). Chitosan was obtained from Carl Roth GmbH + Co. KG. (Germany). Potassium di-hydrogen phosphate, sodium chloride, sodium hydroxide, polyvinyl alcohol (PVA, MW 14 kDa), and glutaraldehyde (Glut) were obtained from Adwic, El-Nasr Pharmaceutical Co. (Egypt). Calcium chloride dihydrate was obtained from Loba Chemie (India). 3-[4,5-dimetylthiazole-2-yl]-2,5-diphenylte-trazolium bromide (MTT) was obtained from Serva (Germany). Dulbecco's modified eagle's medium (DMEM) and penicillin/streptomycin (P/S, 10 IU/ml/10 μg/ml) were purchased from Lonza (Switzerland). Sabouraud dextrose agar (SDA) was obtained from Oxoid Limited (UK). All other chemicals and solvents used were of analytical grade. Roswell Park memorial institute (RPMI) 1640 was obtained from Biowest (USA). All other chemicals and solvents used were of analytical grade.

### Preparation and characterization of atorvastatin-loaded propylene glycol liposomes (ATV/PG-Lip)

#### Formulation of ATV/PG-Lip

Blank PG-Lip were prepared as previously reported [20], with some modifications. Briefly, Lipoid S100 (2 or 4% w/v final concentration) and cholesterol (0 or 0.5% w/v final concentration) were mixed with PG (20% w/v final concentration). Then phosphate buffer (pH 7.4) was added drop wise to the lipid mixture while stirring (IKA Eurostar; IKA Labortechnik, Germany) at 1500 rpm. Stirring was maintained for 30 min at 60 °C.

For the preparation of ATV/PG-Lip, ATV was dissolved in the lipid mixture at different final concentrations (0.2%–0.8% w/v), and the procedure was similarly continued as blank PG-Lip. The prepared formulations were then refrigerated overnight before further analysis.

#### Characterization of ATV/PG-Lip

##### Colloidal properties

The mean vesicle size, polydispersity index (PDI) and zeta potential of formulations were analyzed by dynamic light scattering (DLS) using a Malvern Zetasizer^®^ (Zetasizer^®^ Nano ZS series DTS1060, Malvern Instruments S.A, UK) at a fixed angle (173°) at 25 °C. Before measurements, the samples were suitably diluted by phosphate buffer (pH 7.4).

##### Determination of entrapment efficiency (EE)

The entrapment efficiency (EE) was indirectly estimated by measuring the concentration of free (unentrapped) ATV. Drug-loaded liposomal dispersion (1 mL) was placed in a dialysis bag (Visking^®^, MWCO 12,000–14,000; Serva, Germany) and dialyzed for 2 h against 70 mL of 5% ethanol in phosphate buffer (pH 6.8) at 2–8 °C [20]. ATV in the dialysate was then spectrophotometrically (Agilent Cary 60; Agilent Technologies, USA) determined at λ_max_ of 242 nm [21]. Percentage EE and drug loading (DL; w/w) were then calculated using Eqs. 1 and 2, respectively, where the whole weight was theoretically calculated from the actual weights of used ingredients.

1 $$\mathrm E\mathrm E\%=\frac{\mathrm T\mathrm o\mathrm t\mathrm a\mathrm l\;\mathrm d\mathrm r\mathrm u\mathrm g\;\mathrm a\mathrm m\mathrm o\mathrm u\mathrm n\mathrm t-\mathrm F\mathrm r\mathrm e\mathrm e\;\mathrm d\mathrm r\mathrm u\mathrm g\;\mathrm a\mathrm m\mathrm o\mathrm u\mathrm n\mathrm t}{\mathrm T\mathrm o\mathrm t\mathrm a\mathrm l\;\mathrm d\mathrm r\mathrm u\mathrm g\;\mathrm a\mathrm m\mathrm o\mathrm u\mathrm n\mathrm t}\times100$$

2 $$\mathrm{DL\%\; w}/\mathrm{w}=\frac{\mathrm{Weight \;of \;loaded \;drug}}{\mathrm{Whole \;weight \;of\;ATV}/\mathrm{PG}-\mathrm{Lip }}\times 100$$

##### Transmission electron microscopy (TEM)

Morphology of both blank PG-Lip and ATV/PG-Lip was examined using transmission electron microscopy (TEM; JEM-100S; JEOL Ltd., Japan). Samples were placed onto copper grids and negatively stained with 2% w/v aqueous uranyl acetate solution before examination. Images were captured at X25K magnification at an acceleration voltage of 80 kV.

#### Stability testing

The stability of the selected ATV/PG-Lip formulation was monitored for three months at 2–8 °C. At different time points, the colloidal properties, zeta potential and percentage EE were recorded.

#### In vitro cytocompatibility

Cell compatibility was evaluated using MTT assay [22], with some modifications. Human gingival fibroblasts were cultured in complete culture medium (CCM) containing DMEM supplemented with 10% (v/v) FBS, 1% (v/v) l-glutamine and 1% (v/v) Penicillin–streptomycin. Cells were seeded in 96-well plates at a density of 5 × 10^3^ cell/well and were maintained in CCM. After adherence for 24 h, cells were incubated with plain CCM or treated with ATV solution in DMSO, PG-Lip or ATV/PG-Lip diluted in CCM at different concentrations (corresponding to 1 to 30 μg/mL ATV) and incubated for 48 h. The medium was then discarded, and cells were incubated with MTT solution (0.5 mg/mL in CCM) for 4 h at 5% CO_2_ and 37 °C. Finally, the MTT solution was removed and DMSO was used to dissolve the formed formazan crystals. The absorbance was measured at a wavelength of 570 nm using microplate reader (ELX 800; Biotek, USA). Percentage cell viability was obtained by normalization of optical density values for all groups to the optical density of the control group.

### 3D printing

#### Optimization of ink printability

##### Ink preparation

Inks were prepared using PVA, HPMC, chitosan and PG mixtures. Solutions of PVA and HPMC in phosphate buffer (pH 7.4) were individually prepared, while chitosan was dissolved in 1% acetic acid solution. Afterwards, polymer solutions were blended at different final concentrations and mixed with PG (20% w/v final concentration) to obtain plain ink. Drug-loaded inks were prepared by mixing ATV in PG or ATV/PG-Lip with PVA, HPMC and chitosan at the selected optimum concentrations.

##### Viscosity measurements

Viscosity measurements of the developed inks were evaluated (DV2T viscometer; Brookfield, USA) over the shear rate range of 1.2–3.6 s^−1^ at 25 °C.

##### Measurements of ink-spreading ratio

For evaluation of ink printability, the developed inks were printed into filaments using a 0.5-mm internal diameter nozzle. Then the width of the printed filaments was determined using image analysis (Fiji version 1.52p; National Institutes of Health,USA), and percentage spreading ratio was calculated using Eq. 3 [23].

3 $$\mathrm{Spreading\; ratio\; }\left(\mathrm{\%}\right)=\frac{\mathrm{Measured \;width \;of \;deposited \;filament}}{\mathrm{Internal \;diameter \;of \;the \;nozzle}}\times 100$$

#### 3D printing parameters

The 3D-film structure was created using a 3D-CAD tool (Tinkercad^®^; Autodesk, USA). A square-shaped toroid structure was constructed (dimensions: 10 mm × 10 mm × 2 mm). For investigative analysis, two film designs were developed: monophasic and biphasic (upper- and lower-layer heights of 0.5 mm and 1.5 mm, respectively). Then, using an extrusion-based 3D printer (Robota, Egypt), 3D composite films were plotted in a single step. A syringe with 0.5-mm nozzle diameter was used and printing proceeded at a speed of 3 mm s^−1^ at 25 °C. For maintaining structural integrity, the developed films were crosslinked via in situ layered spraying [19] of Glut (0.25%, 0.5%, 1% or 2% v/v), for crosslinking chitosan in inks [24]. Free glut was neutralized using 0.1 M glycine solution, which was subsequently rinsed with deionized water. 3D-printed films were then dried in a controlled-temperature oven (Memmert GmbH, Germany) at 25 °C for 24 h. Crosslinking was further verified by Fourier transform infrared spectroscopy (FTIR) analysis (Agilent Cary 630; Agilent technologies, USA). Samples were scanned over the range 4000–650 cm^−1^, with a resolution of 2 cm^−1^.

### Characterization of 3D-printed buccal films

#### Scanning electron microscopy (SEM)

The microstructure of the dried 3D-printed films was explored using SEM (100 CX; JEOL, Japan). Before observation, dried films were longitudinally sliced, placed on metal stubs and sputter-coated with gold.

#### In vitro swelling, disintegration and erosion

The fluid intake potential was estimated using percentage swelling. The initial weight (W_0_) of the dried 3D-printed film was recorded using a digital balance. Then, the film was allowed to swell in 5 mL simulated salivary fluid (SSF) (12 mM of potassium dihydrogen phosphate, 40 mM of sodium chloride and 1.5 mM of calcium chloride, pH 6.8) [25]. Experiment was run in a shaking water bath at 37 °C and 50 rpm (Wisebath; Daihan Scientific Co. Ltd, South Korea). At different time intervals, the swollen film weight (W_t_) was determined after carefully removing excess fluid using filter paper. The percentage swelling was calculated using Eq. 4 [19].

4 $$\mathrm{Swelling }\;(\mathrm{\%})=\frac{{\mathrm{W}}_{\mathrm{t}}-{\mathrm{W}}_{0}}{{\mathrm{W}}_{0}}\times 100$$

After reaching maximum swelling, film weight was recorded (W_s_) and film disintegration was monitored by assessing wet film weight (W_e_) over time. The percentage loss in wet weight was calculated using Eq. 5 [26].

5 $$\mathrm{Loss\; in \;wet \;weight }\;\left(\mathrm{\%}\right)=\frac{{\mathrm{W}}_{\mathrm{s}}-{\mathrm{W}}_{\mathrm{e}}}{{\mathrm{W}}_{\mathrm{s}}} \times 100$$

After 8 days, the film was dried in an oven, weighed to determine dry weight (W_d_), and compared to the initial weight of the film (W_0_). The percentage erosion was calculated using Eq. 6 [26].

6 $$\mathrm{Erosion }\;\left(\mathrm{\%}\right)=\frac{{\mathrm{W}}_{0}- {\mathrm{W}}_{\mathrm{d}} }{{\mathrm{W}}_{0}} \times 100$$

#### Residual moisture content

For assessment of the residual moisture content of the developed film, the weight of dried 3D-printed film (W_0_) was determined and recorded. The film was then kept in a desiccator at 25 °C and regularly weighed for 5 days or until a constant weight was obtained (W_t_). The percentage residual moisture content was calculated using Eq. 7 [27].

7 $$\mathrm{Residual \;moisture \;content}\;(\mathrm{\%})=\frac{{\mathrm{W}}_{0}-{\mathrm{W}}_{\mathrm{t}}}{{\mathrm{W}}_{0}}\times 100$$

#### Drug content uniformity

The actual loading of ATV in the developed film was evaluated using non-crosslinked films [18]. After printing, the film was digested in water/methanol (1:1) mixture for complete gel liquefaction and drug extraction. Samples were then filtered and spectrophotometrically analyzed to determine the actual ATV content (W_P_) compared to theoretical drug content (W_T_). The percentage drug content was calculated using Eq. 8 [18].

8 $$\mathrm{Drug \;content }\;(\mathrm{\%})=\frac{{\mathrm{W}}_{\mathrm{P}}}{{\mathrm{W}}_{\mathrm{T}}}\times 100$$

### In vitro drug release

For ATV drug release, dialysis bag technique [20] was applied for ATV/PG-Lip in comparison to ATV solution in PG. Briefly, dialysis bag containing samples (corresponding to 6 mg ATV) was suspended in 70 mL release medium (5% ethanol in phosphate buffer, pH 6.8) to achieve sink condition (previously determined during preliminary solubility studies). ATV release from the developed films was evaluated using total immersion method [18], where films were directly immersed in the release medium with no barrier membrane to achieve sink condition. Experiments were run at 37 °C and 100 rpm in a shaking water bath (Wisebath^®^, UK). At different time points, aliquots were withdrawn for spectrophotometric analysis at 242 nm and were replaced with fresh medium.

### Mucoadhesive properties

For determination of mucoadhesive strength of the developed dried 3D-printed films, texture analysis (texture analyzer CT3; Brookfield, USA) was employed and chicken pouch membranes were used as substrate. The SSF-moistened membrane was affixed to the stationary fixture and the films were sealed to the probe. The texture analyzer probe was lowered to maintain contact with the membrane for 2 min. The force required to separate the adherent film from chicken pouch membrane was recorded for measurement of the mucoadhesive strength and adhesiveness [25].

Mucoadhesion residence time of the developed dried 3D-printed films was assessed using chicken pouch membranes [28]. Briefly, membranes were adhered to the side wall of a glass beaker using cyanoacrylate glue and films were firmly fixed to the membranes with a light force. Membranes were then submerged in SSF and allowed to stir at 150 rpm and 37 °C. Mucoadhesion residence time was indicated as the duration during which the films remained adherent to the membranes before separating off.

### In vitro antifungal activity

#### Determination of minimum inhibitory concentration (MIC)

For MIC determination, agar dilution method was carried out as reported [29], with some modifications. Four different candida strains were used in this assay; *C. albicans* 10231 (fluconazole resistant [30]), *C. albicans* 231GI, in addition to two fluconazole resistant clinical isolates (from urinary tract infection).

SDA agar plates were prepared by mixing molten SDA with serial dilutions of either ATV solution (in DMSO) or ATV/PG-Lip (corresponding to 16–256 µg/mL final ATV concentration). A fresh culture of each of the four candida strains was used to prepare the inoculum. First, the cultures were serially diluted in sterile 0.9% saline to achieve a concentration of 10^4^ CFU/mL. Then, twenty microliters of diluted cultures were spotted on the prepared SDA plates (5 × 10^2^ CFU per spot). SDA agar plates with similar concentrations of fluconazole were prepared in the same way and inoculated with four different isolates to serve as positive control. The plates were incubated at 37 °C for 24 h and the MIC was defined as the least concentration that showed no growth of each strain.

#### Time-dependent antifungal activity

Antifungal activity-time profile was developed as previously described [31], with some modifications, using *C. albicans* ATCC 10231. Reaction mixtures were prepared by mixing a candida inoculum (final count of 5 × 10^5^ CFU/mL in the initial mixture) with ATV solution or ATV/PG-Lip diluted with RPMI (at a final concentration of MIC). Reaction mixtures were then incubated at 37 °C. At different time points, samples were aliquoted, serially diluted in sterile 0.9% saline, plated on SDA plates and incubated for 24 h. Afterward, the detected colonies were counted, and percentage fungal growth was determined. To ensure that there is no carry over from the test formulations that can interfere with colonies growth when plated on SDA, similar mixtures were prepared at a fourfold higher ATV concentration than the applied concentration (32 µg/mL). Samples were aliquoted at zero time point, plated and the count was found to be similar to the control mixture (with no test formulations) indicating the absence of any carryover effect.

#### Agar diffusion assay

Agar diffusion assay was employed using Mueller–Hinton agar plates supplied with 2% dextrose, 0.5 µg/mL methylene blue dye, as recommended by the CLSI yeast susceptibility testing protocol [32]. The agar plates were streaked in three different directions using a swab dipped into *C. albicans* ATCC 102,231 (10^6^ CFU/mL). A cork-borer was used to cut 7-mm pores in the agar, then pores were loaded with the tested samples (corresponding to 250 µg ATV).

#### Scanning electron microscope study

The ultrastructural morphology of the candida cells was investigated using SEM as described [33], with some modifications. *C. albicans* 10231 was grown overnight in nutrient broth, adjusted to 10^6^ CFU/mL and then incubated either alone (control *C. albicans* 10231 cells) or treated with ATV solution or ATV/PG-Lip (at a final concentration of MIC) for 2 h at 37 °C and 100 rpm in a shaking incubator. After incubation, the samples were centrifuged at 6000 rpm for 10 min, then fixed with 4% formaldehyde and 1% glutaraldehyde in phosphate-buffered saline, pH 7.2. Cells were further treated with ethanol before gold-coating and examination.

### In vivo antifungal activity

#### Induction of oral candidiasis 

The Institutional Animal Care and Use Committee (IACUC), Alexandria University, Egypt (AU0620215231101), approved the in vivo animal study. All experiments were conducted in accordance with the ethical criteria of the European Parliament Directive 2010/63/EU. In vivo induction of oral candidiasis was carried out as previously reported [34], with slight modifications. Briefly, 15 healthy albino rabbits (1.7 ± 0.2 kg) were chosen for in vivo investigations, where 3 animals were maintained without infection (healthy control group). Before induction of infection, swabs were collected from the buccal mucosa, palate and tongue of animals under anesthesia (7 mg/kg xylazine and 40 mg/kg ketamine hydrochloride). Swabs were then plated on SDA plates to check for any oral fungal infections, only non-infected animals were included in the study. For preparation of the inoculum used for infection, *C. albicans *ATTC 10231 was grown in nutrient broth and diluted in PBS to achieve 10^6^ CFU/mL. Induction of oral candidiasis was done under anesthesia by swabbing the oral cavity (buccal mucosa, palate and tongue) with the prepared inoculum once daily for six days. Successful oral candidiasis induction was confirmed by oral swabs. Afterwards, infected animals were allocated to 4 more groups (n = 3), either treated with films containing ATV, ATV/PG-Lip or PG-Lip or left untreated (infected/untreated control). At the end of the treatment period (5 days), animals were euthanized by an overdose of anesthesia, and the oral cavity tissues were collected.

#### Enzyme-linked immunosorbent assay (ELISA) of inflammatory biomarkers

After sacrifice, collected tongue tissues were frozen and stored at -80 °C. Tissue homogenates (10% w/v) were then prepared by homogenization (Ultra Turrax; IKA Labortechnik, Germany) of extracted tissues in ice-cold PBS. Mixtures were then centrifuged (3 K-30; Sigma, Germany) at 10,000 rpm and 4 °C for 10 min. Supernatants were collected for determination of tumor necrosis factor-alpha (TNF-α) (Cat# CSB-E06998Rb, Cusabio, USA) and interleukin-6 (IL-6) (Cat# CSB-E06903Rb, Cusabio, USA) levels, according to the manufacturer’s instructions.

#### Histological and histomorphometric analysis

After sacrifice, collected tissue samples were preserved in 10% formalin for histological examination. Specimens from different mucosal sites (buccal and tongue) were stained with specific stain Periodic acid-Schiff (PAS) to detect the fungal hyphae and to assess the infectivity and inflammation score. Whereas cheek and tongue specimens of healthy control group were processed for comparison.

For morphometric analysis, five microscopical fields were captured by a digital camera (Olympus DP20) connected to microscope (Olympus BX41) at × 200 and × 400 magnifications. Scoring of fungal infection and inflammation proceeded as follows: score 0 for the absence of hyphae/spores and inflammation; score 1 for the presence of hyphae/spores in the upper mucosal one-third and 1–3 intraepithelial micro-abscesses; score 2 for the presence of hyphae/spores in the upper epithelial two-third with 4–6 micro-abscesses within the mucosal layer and sporadic inflammatory infiltrate in the lamina propria; score 3 for hyphae/spores present in the whole mucosal layer with > 6 micro-abscesses or large abscess formation in the epithelial layer and diffuse submucosal inflammatory infiltrate [35].

### Statistical analysis

Data was statistically analyzed using analysis of variance (ANOVA) followed by Duncan’s post-hoc pairwise comparisons, whenever needed, using SPSS 20.0; (SPSS Inc., Chicago, IL, USA). Experiments were carried out at least in triplicate, applying at least three different samples, and data are expressed as mean ± standard deviation. Whereas candida/inflammation scoring is expressed as median values. p values ≤ 0.05 are considered significant.

## Results and discussion

### Colloidal properties of ATV/PG-Lip

For optimization of ATV/PG-Lip colloidal properties, the effect of Lipoid S100, cholesterol and ATV content was studied (Table 1). A significant (p ≤ 0.05) decrease in liposomal vesicle size was observed on reducing Lipoid S100 concentration from 4% w/v (F1; 3922 ± 176.2 nm) to 2% w/v (F2; 760.3 ± 18.4 nm) with no significant effect (p > 0.05) on PDI. Similarly, a decrease in average vesicle size from 913 ± 35 nm to 268 ± 15.2 nm was previously reported with lowering phospholipid content from 900 to 300 mg [36]. Addition of cholesterol to the lipid mixture resulted in further significant (p ≤ 0.05) decrease in both vesicle size and PDI, as evident from comparing 0% w/v cholesterol (F2; 760.3 ± 18.4 nm and PDI 0.46 ± 0.01) to 0.5% w/v cholesterol (F3; 430.7 ± 13.8 nm and PDI 0.24 ± 0.001), reflecting improvement in the homogeneity of liposomal populations. These results are in agreement with previous findings showing reduction in particle size and PDI upon inclusion of cholesterol [37]. Based on its favorable colloidal properties, F3 (2% w/v Lipoid S100 and 0.5% w/v cholesterol) was selected for drug loading and further characterization.

**Table 1:** Formulation and optimization parameters of ATV/PG-Lip

**Formulation code**	**Formulation parameters**^a^			**Results**			
	**Lipoid S100 (%w/v)**	**Cholesterol (%w/v)**	**ATV** **(%w/v)**	**Size (nm)**	**PDI**	**EE%**	**DL (%w/w)**
F1	4	-	-	3922 ± 176.2	0.43 ± 0.01	-	-
F2	2			760.3 ± 18.4	0.46 ± 0.01		
F3		0.5		430.7 ± 13.8	0.24 ± 0.001		
F4			0.2	236.5 ± 16.9	0.29 ± 0.01	83.4 ± 0.2	7.06 ± 0.25
F5			0.4	250 ± 19.5	0.26 ± 0.01	86.45 ± 1.34	13.5 ± 0.16
F6 (selected formulation)			0.6	223.3 ± 2.1	0.12 ± 0.001	81.15 ± 1.88	18.33 ± 2.16
F7			0.8	307.9 ± 2.5	0.37 ± 0.01	54.21 ± 4.9	16.48 ± 1.53

^a^All formulations contained PG (20% w/v)

Compared to F3 (430.7 ± 13.8 nm), ATV loading (F4–F7) resulted in a significant (p ≤ 0.05) reduction in vesicle size irrespective of the percentage of ATV loading. This could be attributed to ATV entrapment mainly in the liposomal lipid bilayer. This is due to ATV lipophilic nature [38] and slight solubility in phosphate buffer (pH 7.4), which consequently reduces repulsive forces between phosphate groups of lipid molecules leading to closer packing [20]. Similar size reduction has been previously reported upon lipophilic drug loading into liposomes for miconazole [20] and diclofenac [39]. Increasing ATV concentration from 0.2 to 0.6%w/v did not significantly (p > 0.05) affect vesicle size. However, a slight but statistically significant (p ≤ 0.05) increase in size was observed upon loading of 0.8%w/v ATV (F7; 307.9 ± 2.5 nm).

The average zeta potential for ATV-loaded formulations (F4–F7) was -18 ± 0.2 mV, similar to previously developed proposomes [13]. The negative charge observed is likely due to phosphate group ionization [40].

### ATV entrapment efficiency

As can be seen in Table 1, ATV initial employed concentrations of 0.2–0.6% w/v resulted in percentage EE exceeding 80%. This was accompanied with a significant (p ≤ 0.05) increase in percentage DL on increasing ATV from 0.2% w/v (F4; 7.06 ± 0.25% w/w) to 0.6% w/v (F6; 18.33 ± 2.16% w/w). It is worth noting that percentage DL of ATV-loaded liposomes prepared by thin film hydration sonication method was 4.01 ± 0.05% w/w, as previously reported [38]. The comparatively enhanced ATV loading capacity observed in the current study could be attributed to ATV high solubility in PG (175.99 ± 2.08 mg/mL [41]) and further confirms PG-Lip as nano vector of high loading potential for ATV delivery.

**Fig. 1:**
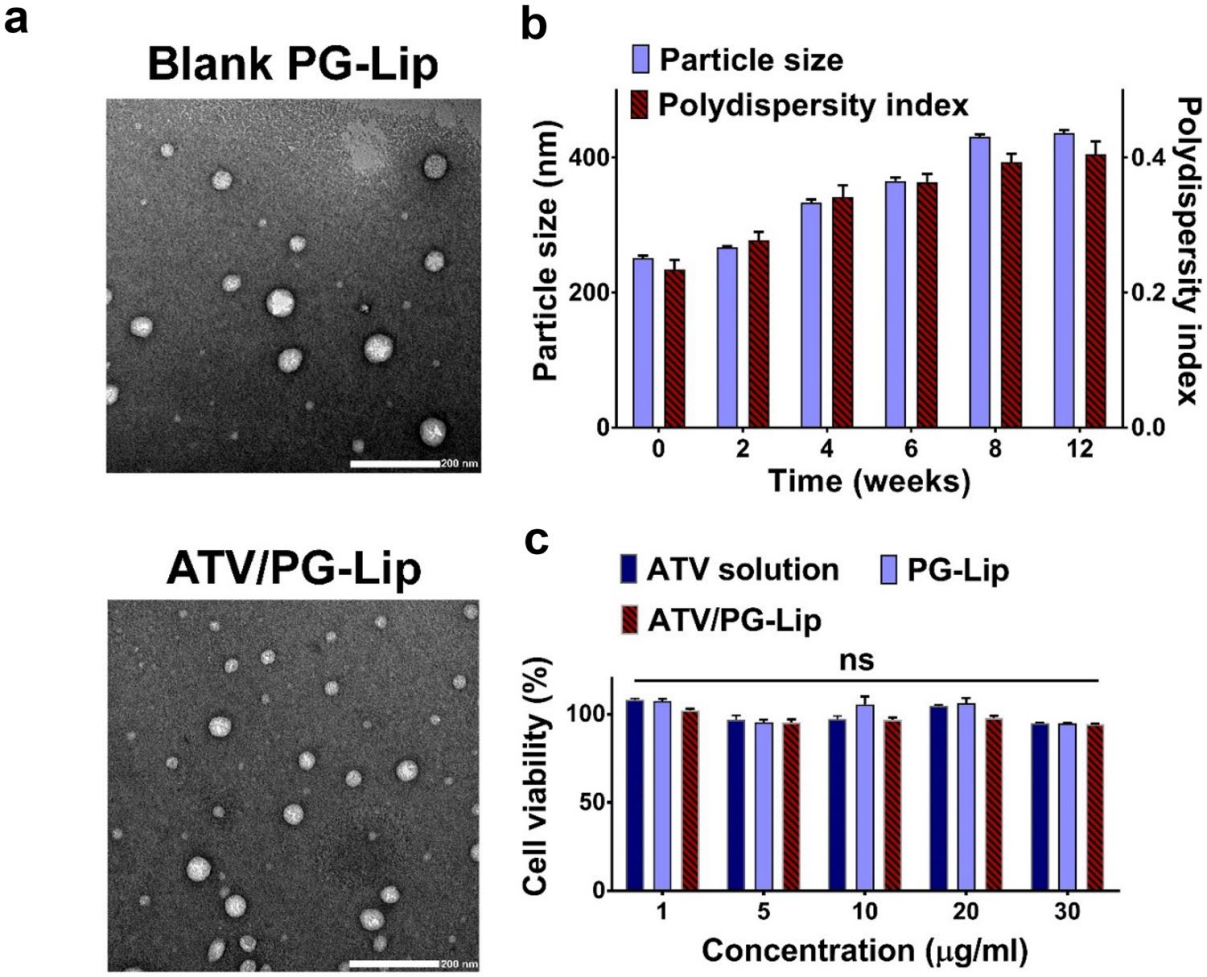
Characterization of the developed ATV/PG-Lip (**a**–**c**). Transmission electron microscopy images showing the morphology of blank PG-Lip and ATV/PG-Lip (**a**), scale bar = 200 nm. Physical stability data of ATV/PG-Lip based on vesicle size and polydispersity index when stored at 4 °C for 12 weeks (**b**), n = 3 at p ≤ 0.05. Cell cytocompatibility study for different formulations on human gingival fibroblasts (**c**), n = 7. Data represents mean ± SD. ns: statistically nonsignificant difference at p ≤ 0.05

However, by further increasing ATV initial concentration above 0.6% w/v, there was a significant (p ≤ 0.05) decrease in percentage EE (F7; 54.21 ± 4.9% w/v) with an insignificant (p > 0.05) change in percentage DL (F7; 16.48 ± 1.53% w/w; Fig. 1S), reflecting a maximum average percentage DL of ATV in PG-Lip of 17.41 ± 0.93% w/w. It should be stated that upon further increase in ATV concentration to 1% w/v, drug precipitation was evident, and it was hence excluded from the study.

Accordingly, F6 was selected as the optimized ATV-loaded formulation with optimum liposomal vesicle size (223.3 ± 2.1 nm), PDI (0.12 ± 0.001), and maximum percentage DL (18.33 ± 2.16% w/w) and was henceforth referred to as ATV/PG-Lip.

### Transmission electron microscopy (TEM)

The morphology for both PG-Lip and ATV/PG-Lip was microscopically investigated. Both liposomal formulations showed spherical structure, with uniform vesicle size (Fig. 1a).

It is worth noting that microscopically determined vesicle size for PG-Lip and ATV/PG-Lip were lower than values determined using DLS. This difference in vesicle size was previously reported [42] and was attributed to the variation in sample preparation between TEM and DLS techniques. Since sample processing for TEM involves drying of the deposited dispersion on the grid prior to examination, subsequent water evaporation and possible shrinkage of the vesicles could occur. Also, TEM facilitates the examination of single liposomal vesicle, avoiding possible agglomeration.

### Stability testing

The effect of storage on colloidal properties and ATV percentage EE of ATV/PG-Lip was monitored over 3 months at 4 °C (Fig. 1b). At the two-week interval, no change in either particle size or PDI was observed. A gradual increase in both parameters was noted thereafter, reaching a vesicle size of 436.5 ± 4.66 nm and PDI of 0.405 ± 0.018 by the end of the storage period (p ≤ 0.05), as previously reported [43], and could be attributed to vesicular aggregation. Regarding zeta potential, no significant (p > 0.05) change was observed after 3 months (-18.2 ± 0.3 mV).

Furthermore, successful ATV entrapment was maintained over 3 months, where the percentage EE remained above 80%. The potential of ATV/PG-Lip to efficiently retain ATV over time presents PG-Lip as a promising delivery system of reasonable stability overcoming a major drawback of liposomal systems which is encapsulated drug leakage [44].

### In vitro cell cytocompatibility

For the investigation of cytocompatibility, the effect of PG-Lip and ATV/PG-Lip on the cell viability of human gingival fibroblasts was evaluated using MTT assay. Formulations were tested and compared to ATV solution at concentrations (1–30 µg/mL).

As seen in Fig. 1c, the results revealed that there was no significant (p > 0.05) reduction in cell viability for all tested formulations across different concentrations, verifying the cytocompatibility of the developed formulations.

### 3D printing

#### Optimization of ink printability

##### Morphological assessment

Different concentrations of polymer blend (PVA, HPMC, and chitosan; Table 2) were employed to optimize plain ink composition. PG (20% w/v final concentration) was used for plain ink preparation, as similarly included within liposomal formulation. By visually examining filaments continuity during extrusion, the printability of various inks was qualitatively assessed.

**Table 2:**
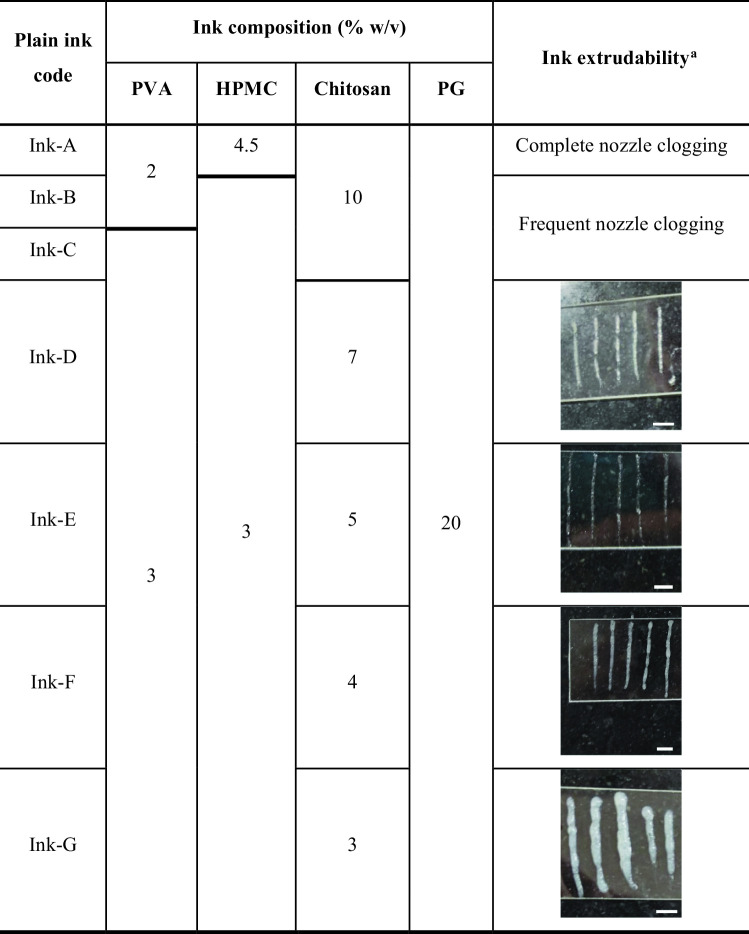
Formulation and morphological assessment of plain inks for 3D printing optimization

^a^Scale bar = 5 mm

The ink extrudability was improved by decreasing HPMC concentration from 4.5% w/v (ink A) to 3% w/v (ink B), where ink A showed extrusion failure resulting in complete nozzle clogging.

By increasing PVA concentration from 2% w/v (ink B) to 3% w/v (ink C), the ink showed less frequent nozzle clogging and less resistance to extrusion. This is in line with a previous study [19], where incorporating PVA and hyaluronic improved the extrudability of plain gelatin ink.

Additionally, the employed chitosan concentration greatly affected ink printability. By reducing chitosan concentration from 10% w/v (inks A–C) to 7% w/v (ink D), extrusion was relatively improved, however the ink formed a gritty non-continuous filament. Whereas decreasing chitosan concentration to 5 and 4% w/v (inks E and F, respectively) demonstrated more efficient extrusion, resulting in smooth continuous filaments. On the other hand, 3% w/v chitosan (ink G) formed a looser filament.

Accordingly, inks containing 10% w/v chitosan (inks A–C) were exempted from further trials and more experimental characterization was carried out for plain ink optimization using plain inks D–G (3% w/v PVA, 3% w/v HPMC and 7–3% w/v chitosan).

##### Viscosity measurements

**Fig. 2:**
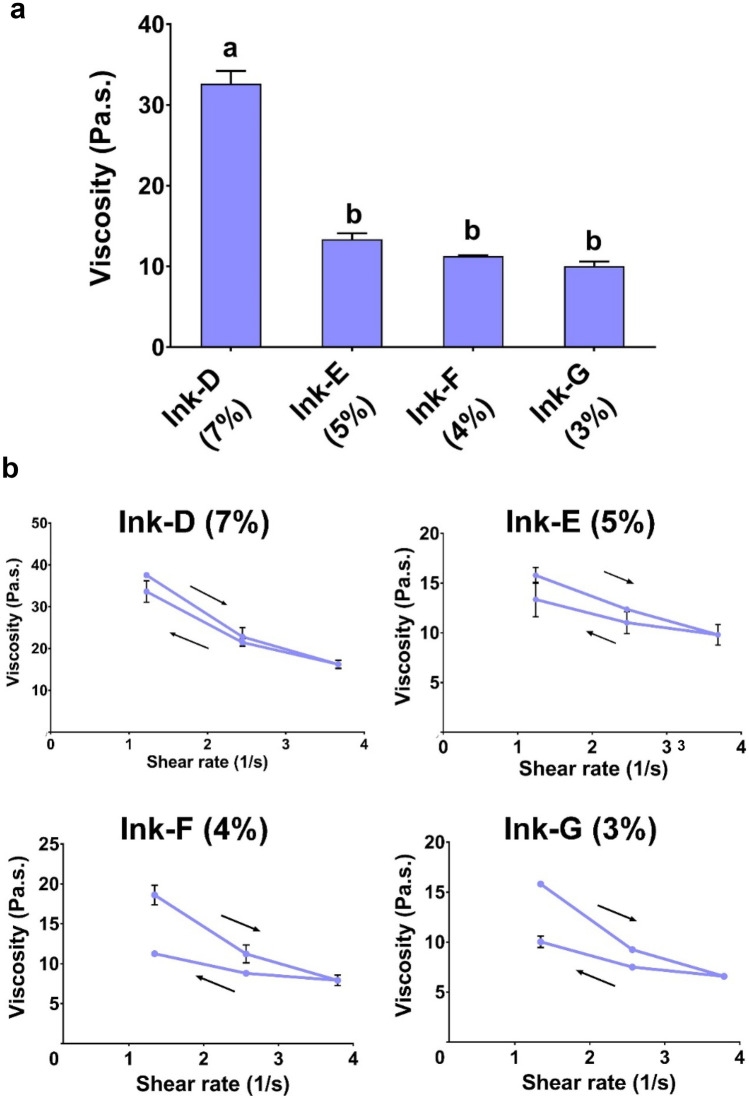
Viscosity measurement of the developed inks for 3D printing (**a**, **b**). Initial viscosity values (at 1.2 s^−1^ shear rate) of tested plain polymer inks containing 3% w/v PVA and 3% w/v HPMC using different chitosan concentrations (3–7% w/v) (**a**). Data indicates mean ± SD, n = 3. Bars bearing different letters indicate statistically significant difference: a > b, at p ≤ 0.05. Viscosity values (at different shear rates) of tested plain inks containing 3% w/v PVA and 3% w/v HPMC using different chitosan concentrations and corresponding hysteresis loops of plain polymer inks; ink-D (7%), ink-E (5%), ink-F (4%) and ink-G (3%) (**b**)

In extrusion-based 3D printing, adjusting ink viscosity is critical for optimum extrusion without nozzle clogging and maintaining shape fidelity after printing [19]. Also, shear thinning property is a requirement for a continuous flow during 3D printing procedure [19]. In our study, characterization of the prepared inks was conducted via viscosity measurements. Initial viscosity values (at shear rate 1.2 s^−1^) were measured for plain inks containing decreasing chitosan concentrations (7–3% w/v; inks D–G) as demonstrated in Fig. 2a.

Results showed that 7% w/v chitosan (ink D) demonstrated statistically (p ≤ 0.05) highest initial viscosity (32.63 ± 1.56 Pa.s.). This finding was reflected in the poor printability of ink D, forming non-continuous filament upon extrusion. However, there was no significant (p > 0.05) difference between initial viscosity values for 5–3% w/v chitosan (inks E–G), with an average value of (11.55 ± 1.4 Pa.s.).

All the tested plain inks (D–G) demonstrated a noticeable shear thinning behavior, as evident from viscosity vs stress curves (Fig. 2b), where the viscosity decreased by increasing the shear rate from (1.2 to 3.6 s^−1^; down-curve). Also, on decreasing shear rate (3.6 to 1.2 s^−1^; up-curve), viscosity values recovered gradually, generating a hysteresis loop and reflecting a thixotropic behavior [45]. Similarly, it was previously reported that chitosan gels possessed a shear thinning property [46]. By comparing hysteresis loops of inks E and F, it can be seen that both down- and up-curves were closer for ink E, implying more efficient and rapid viscosity recovery on release of shear [45]. Rapid recovery is desirable for achievement of high precision and shape fidelity of the printed structure.

Accordingly, ink E (3% w/v PVA, 3% w/v HPMC and 5% w/v chitosan) was selected as optimum plain ink and was henceforth referred to as plain polymer ink. Drug-loaded inks were prepared by mixing ATV in PG or ATV/PG-Lip with PVA (3% w/v), HPMC (3% w/v) and chitosan (5% w/v) and were referred to as ATV@ink and ATV/PG-Lip@ink, respectively. As control, PG-Lip@ink was similarly prepared using PG-Lip. Shear-thinning viscosity and elastic behavior were also verified for all the optimized inks (Fig. 2S).

##### Spreading ratio

Spreading ratio is considered as an important parameter to optimize ink printability. For production of highly precise hydrogel structures, lower spreading ratios are preferred [23]. In this study, we recorded percentage spreading ratio for the optimized inks (5% w/v chitosan); plain polymer ink, ATV@ink, PG-Lip@ink, ATV/PG-Lip@ink. For comparative analysis, spreading ratio was investigated for inks prepared using lower chitosan concentration (4% w/v chitosan); plain polymer ink F, ATV-, PG-Lip- and ATV/PG-Lip-loaded inks.

**Fig. 3:**
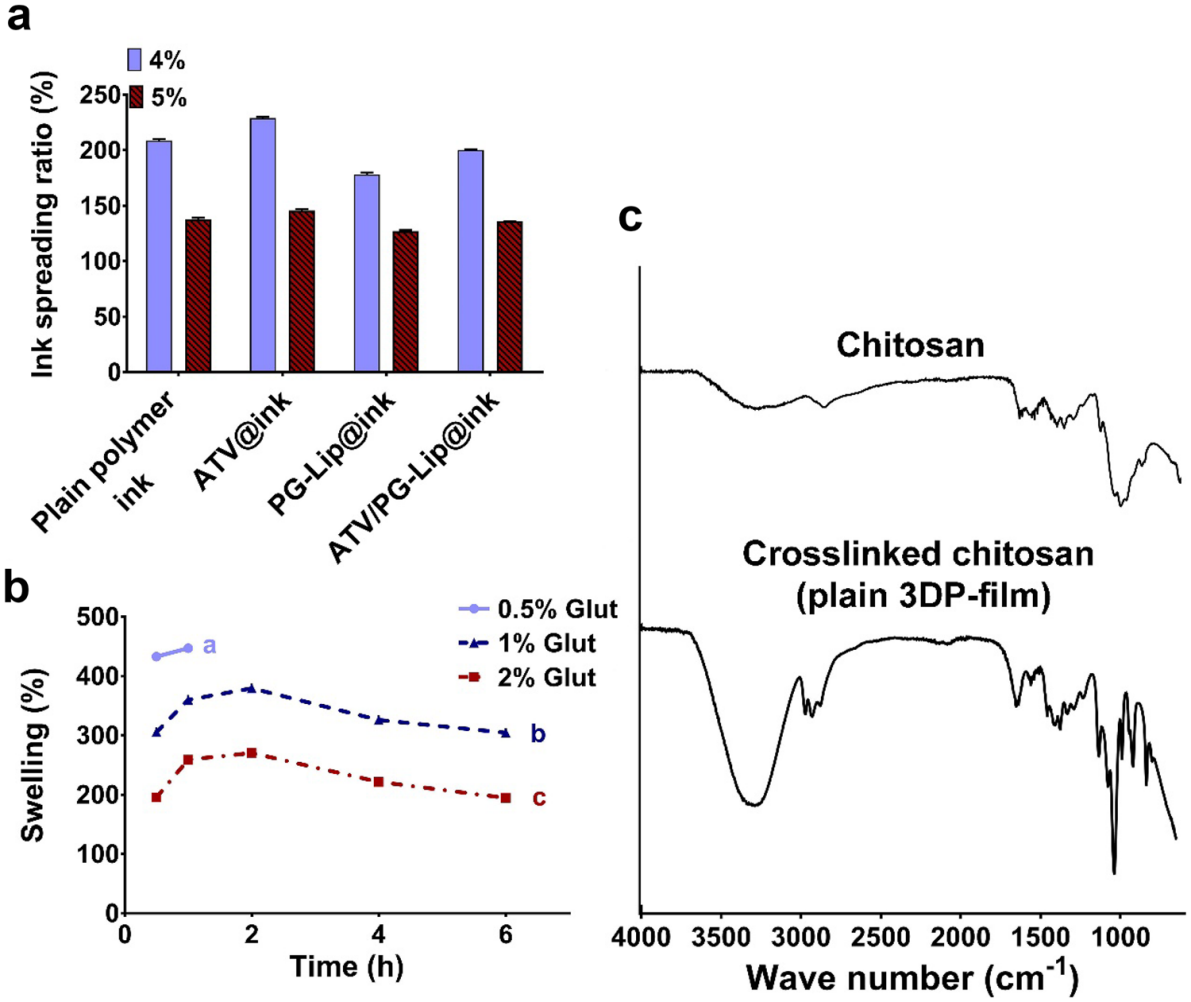
Optimization of 3D printing parameters (**a**–**c**). Optimization of ink spreading ratio for different tested inks containing 3% w/v PVA and 3% w/v HPMC, using 4% and 5% w/v chitosan (**a**). Data indicates mean ± SD, n = 3, different letters indicate statistically significant difference: a > b > c, at p ≤ 0.05. Effect of glutaraldehyde concentration on percentage swelling of ATV@3DP-film (**b**). FTIR spectra of chitosan powder and plain 3DP-film (**c**), verifying efficient chitosan crosslinking by glutaraldehyde

Interestingly, inks containing 4% w/v chitosan presented significantly (p ≤ 0.05) higher percentage spreading ratio than their respective counterparts as shown in Fig. 3a. This finding is due to the rapid viscosity recovery demonstrated by ink E (5% w/v chitosan) compared to ink F (4% w/v chitosan) and further justifies the opportune selection of Ink E. Moreover, results showed no significant (p > 0.05) difference in percentage spreading ratios for different optimized inks with 5% w/v chitosan (Fig. 3a).

Collectively, plain polymer ink, ATV@ink, PG-Lip@ink, ATV/PG-Lip@ink were optimally prepared using 3% w/v PVA, 3% w/v HPMC and 5% w/v chitosan.

#### Crosslinking 

##### Optimization of crosslinker concentration

Structural strength is important to develop constructs with sufficient durability, which depends mainly on crosslinking [19]. In our work, we used Glut as an effective chitosan crosslinker [24]. For optimization of Glut concentration, monophasic square-shaped toroid constructs were 3D printed applying plain polymer ink and were crosslinked using 0.25, 0.5, 1 or 2% v/v Glut solutions. 3D-printed constructs were then dried before evaluation of film swelling over 6 h.

For 0.25% v/v Glut, the tested films were completely eroded/disintegrated after 0.5 h (not shown in Fig. 3b), while the film prepared using 0.5% v/v Glut lost integrity after 1 h (Fig. 3b). However, for both 1% v/v and 2% v/v Glut, structural integrity was maintained, and films swelling was noted over 6 h. Therefore, for higher safety and biocompatibility, the lower Glut concentration (1% v/v) was selected as optimum and was applied in this study.

##### Fourier transform infrared spectroscopy (FTIR)

For verification of efficient crosslinking of chitosan functional groups, FITR was conducted for uncrosslinked chitosan powder in comparison to crosslinked chitosan (in a monophasic construct printed using plain polymer ink).

As shown in Fig. 3c, uncrosslinked chitosan exhibited a characteristic broad band at 3324 cm^−1^, collectively due to the stretching vibrations of the OH and the functional NH_2_ groups in chitosan. The polysaccharide structure of chitosan was indicated by bands at 1373, 1021, and 2876 cm^−1^, related to stretching vibrations of C-N, C-O, and C-H, respectively [47]. The primary amine N–H bond bending is represented by the band seen at 1576 cm^−1^. The C = O stretching vibration in the amide group, produced by the incomplete deacetylation of chitin, is assigned to the band at 1648 cm^−1^ [47].

For crosslinked chitosan in film (Fig. 3c), a stretching band around 1642 cm^−1^ can be seen, which is of higher intensity than the similarly located C = O vibration band at 1648 cm^−1^ for uncrosslinked chitosan. This band corresponds to the crosslinking-typical imine bond (C = N), probably resulting from the crosslinking reaction between Glut and chitosan amino groups [47]. Also, a relative increase in the 2875 cm^−1^ band intensity can be related to the C-H crosslinked bond, probably overlapping with the -CH_2_- groups in the Glut structure [47]. Collectively, these findings confirm the efficient crosslinking in the ink polymer matrix, further validating the adopted crosslinking technique.

#### 3D printing of composite mucoadhesive buccal films

We developed a monophasic square-shaped toroid film structure with dimensions: 10 mm × 10 mm × 2 mm. We further developed a biphasic design (upper- and lower-layer heights of 0.5 mm and 1.5 mm, respectively), for investigative analysis. The toroid architecture was selected for designing the buccal film, to allow more favourable circulation of buccal fluids, film interaction and drug release. Monophasic composite films were obtained via 3D printing of plain polymer ink, ATV@ink, PG-Lip@ink and ATV/PG-Lip@ink then drying at controlled temperature of 25 °C to obtain plain 3DP-film, ATV@3DP-film, PG-Lip@3DP-film and ATV/PG-Lip@3DP-film, respectively. Whereas the biphasic 3DP-film was printed using ATV@ink for upper layer and ATV/PG-Lip@ink for lower layer.

**Fig. 4:**
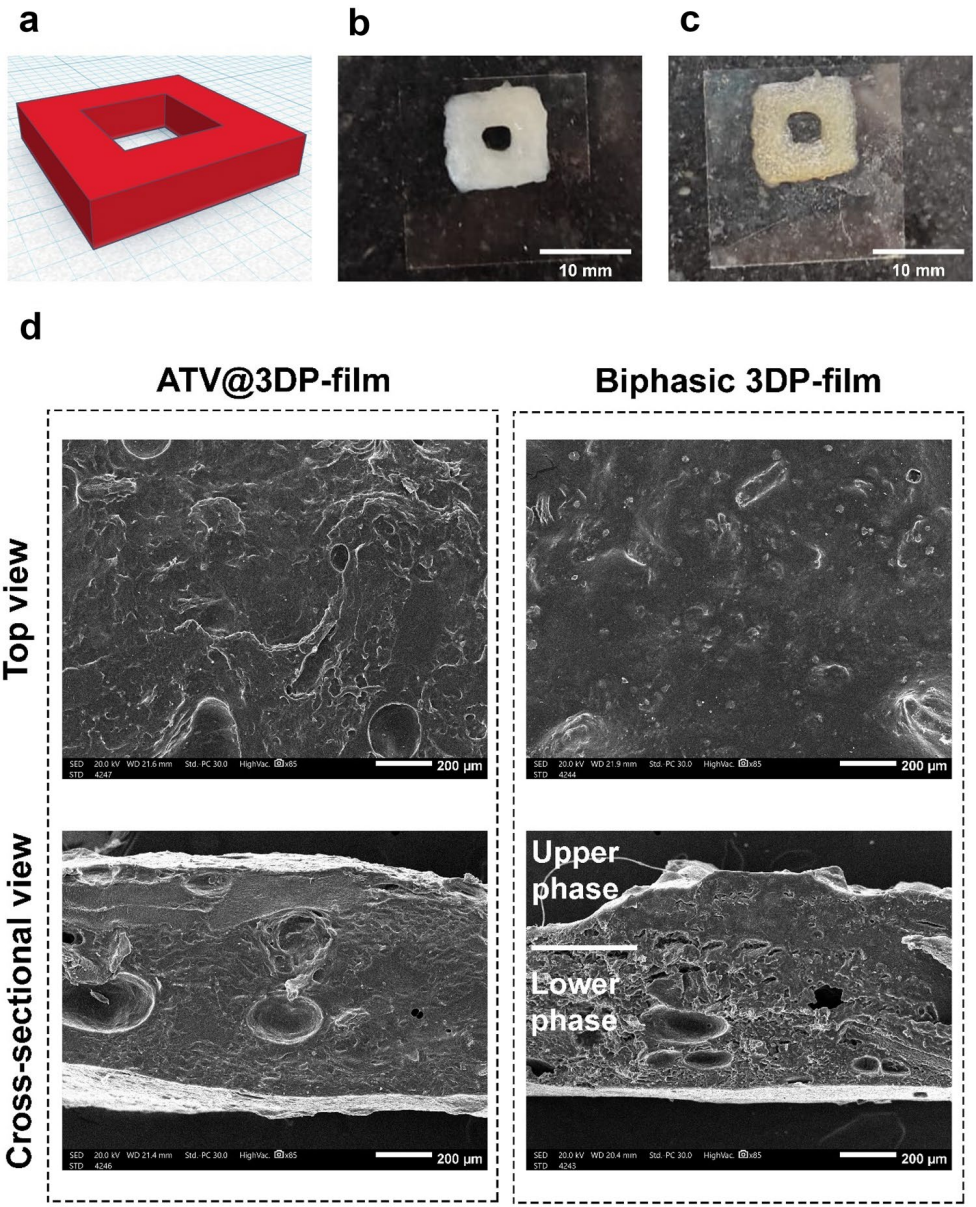
3D printing of mucoadhesive buccal films (**a**–**d**). 3D computer-aided design of monophasic ATV/PG-Lip@3DP-film with dimensions 10 mm × 10 mm × 2 mm (**a**). Representative image of freshly prepared (**b**) and dried (**c**) ATV/PG-Lip@3DP-film. Scale bar = 10 mm. Scanning electron micrographs of the developed 3DP-films (**d**), illustrating top view and cross-sectional view of ATV@3DP-film, showing a relatively homogenous phase. Whereas the top view and cross-sectional view of biphasic 3DP-film show different microstructure for upper and lower phases. Scale bar = 200 µm

As shown in Fig. 4a–c, extrusion-based 3D printing provided accurate production of the tested CAD geometry. We further investigated the microstructure of monophasic ATV@3DP-film and biphasic 3DP-film using SEM. Cross sectional view of the monophasic ATV@3DP-film (Fig. 4d) showed a dense, compact structure of the applied polymeric matrix, further indicating structural integrity and efficient component homogeneity. On the other hand, cross sectional view of the biphasic film revealed distinctive upper and lower layers, where the upper layer reflected smooth compactness. Whereas the lower layer presented more porosity and matrix roughness, which might be attributed to the presence of liposomes. The clear demarcation of the upper and lower layers further verifies the reliability of the adopted 3D printing technique.

### Characterization of the 3D-printed buccal films

The developed dried, composite films were characterized regarding swelling and time-driven disintegration based on assessing water uptake followed by monitoring change in swollen wet film weight. Film erosion was assessed based on final dry film weight. Additionally, residual moisture content and drug content uniformity were determined.

#### Swelling, disintegration and erosion 

The degree of swelling of polymer blend is a critical parameter affecting film mucoadhesion due to detachment and relaxation of polymer chains occurring upon swelling [48].

**Fig. 5:**
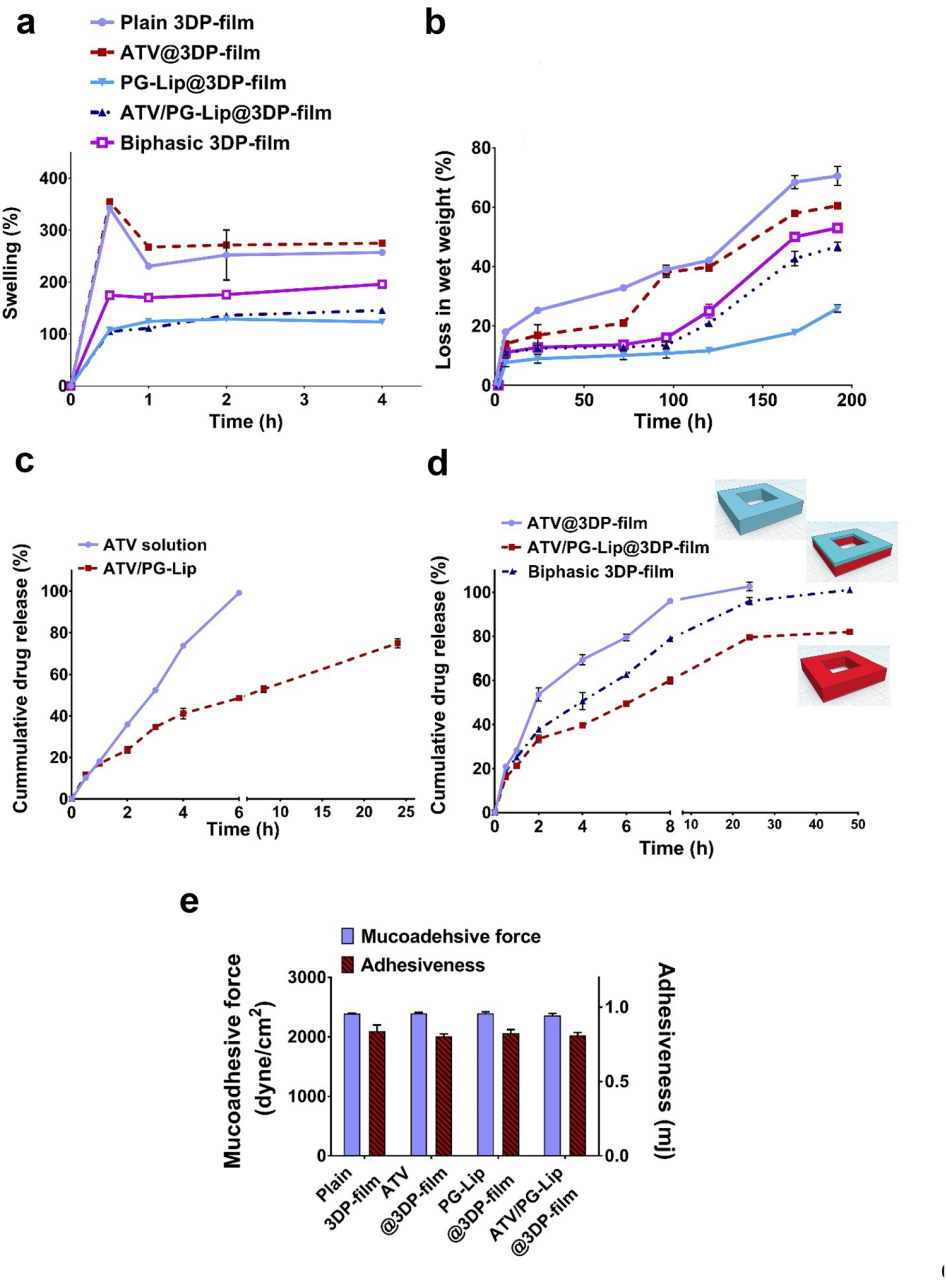
Characterization of the developed 3D printed films (**a**–**e**). Swelling (**a**) and loss in maximum swollen wet weight over time (**b**) of the developed 3DP-films. In vitro cumulative release profile for ATV solution and ATV/PG-Lip using the dialysis bag technique (**c**) and ATV@3DP-film, ATV/PG-Lip@3DP-film and biphasic film using the total immersion method (**d**). Release experiments were conducted at 37 °C in 5% ethanol phosphate buffer, pH 6.8. Mucoadhesive force and adhesiveness of different films (**e**). Data indicates mean ± SD, n = 3, p ≤ 0.05

Percentage swelling of monophasic plain 3DP-film, ATV@3DP-film, PG-Lip@3DP-film and ATV/PG-Lip@3DP-film in SSF is shown in Fig. 5a. Percentage swelling values for both plain 3DP film and ATV@3DP-film were significantly (p ≤ 0.05) higher than liposomal films (PG-Lip@3DP-film and ATV/PG-Lip@3DP-film). The effect of nanoparticles on swelling of polymeric matrices has been previously reported [18], and could be attributed to the physical crosslinking that is created between polymeric chains in the presence of liposomal vesicles, creating more compact and tighter structures. Also, the reduced swelling of liposome loaded films could be attributed to the lower hydrophilicity of liposomes compared to the polymeric matrix [49]. It is worth noting that initial swelling for both plain 3DP-film and ATV@3DP-film (342.4 ± 0.6% and 354.6 ± 1.4% at 0.5 h, respectively) was followed by an apparent decline in water uptake (230.50 ± 1.45% and 267.35 ± 2.6% at 1 h, respectively). This finding could possibly be partly referred to the diffusion of PG from the swollen films into the surrounding medium and consequent reduction of films weight. Nevertheless, this behavior was not observed for liposomal films, probably because of the relative partial confinement of PG within the liposomes in films. In addition, the longer diffusion path provided by liposomes [49] might have hindered entrapped PG diffusion, resulting in constant swelling behavior. For biphasic 3DP-film, percentage swelling values followed an intermediate pattern between ATV@3DP-film and ATV/PG-Lip@3DP-film.

As shown in Fig. 5a, films could undergo swelling, maintaining structural integrity for 4 h. Following swelling, films started disintegrating due to gel liquefaction which was reflected by wet weight loss. Percentage wet weight loss was recorded for different films (Fig. 5b), where films exhibited an increase in wet weight loss when compared to the maximum swollen weight due to disintegration. Both plain 3DP-film and ATV@3DP-film showed higher percentage wet weight loss (70.5 ± 3.2% and 60.8 ± 1.2% after 192 h, respectively) than liposomal films (25.8 ± 1.2% and 46.7 ± 1.5% for PG-Lip@3DP-film and ATV/PG-Lip@3DP-film, respectively). The higher weight loss for ATV/PG-Lip@3DP-film compared to PG-Lip@3DP-film can be possibly referred to ATV release. It is possible that ATV release has created over time more channels for the diffusion of the surrounding medium and consequently higher disintegration and gel liquefaction. The pattern for biphasic films wet weight loss similarly proceeded as detected for percentage swelling. This result was in line with the lower percentage swelling results for liposomal films, further pointing out the possible physical crosslinking.

Finally, percentage film erosion was determined after immersion for 8 days in SSF. Percentages loss in dry film weight for plain 3DP-film and ATV@3DP-film were 89 ± 0.6% and 87 ± 0.9% respectively. However, for PG-Lip@3DP-film and ATV/PG-Lip@3DP-film, corresponding percentages were 78 ± 1.2% and 76 ± 0.3% respectively.

#### Residual moisture content

Residual moisture content is an important parameter in buccal films due to its obvious impact on their stability against microbial infections [50].

The average moisture content for all tested films (plain 3DP-film, ATV@3DP-film, PG-Lip@3DP-film and ATV/PG-Lip@3DP-film) was 6.57 ± 0.75% w/w.

#### Drug content uniformity

Percentage drug content was utilized as an indicator of content homogeneity to measure printing accuracy and determine the effectiveness of process parameters for developing tailorable drug-loaded films with acceptable reproducibility [18].

Drug content values for ATV@3DP-film and ATV/PG-Lip@3DP-film were 96.9 ± 5.7% and 95.2 ± 0.75%, respectively, which indicated that the drug is uniformly contained in films, further validating the adopted printing procedure.

### In vitro drug release

Drug release was investigated for ATV/PG-Lip compared to ATV solution using dialysis bag technique. Whereas drug release from ATV@3DP-film and ATV/PG-Lip@3DP was tested using total immersion method. For investigative analysis, drug release from biphasic 3DP-film was included.

As seen in Fig. 5c, ATV/PG-Lip presented a significantly (p ≤ 0.05) lower percentage release (48.6 ± 1.2%) compared to ATV solution (100 ± 0.3%) within 6 h. The complete diffusion of ATV from solution verifies the drug dialyzability and establishes the controlled drug release from ATV/PG-Lip. The kinetic analysis of release profiles (as detailed in Table 1S) showed that drug release from ATV/PG-Lip fitted Higuchi model, possibly implying ATV diffusion from liposomal bilayer.

Drug release study from the developed films demonstrated that ATV release rate for all time points increased in the following pattern: ATV@3DP-film > biphasic 3DP-film > ATV/PG-Lip@3DP-film (Fig. 5d). More specifically, approximately 50% ATV release was achieved after around 2, 4 and 6 h for ATV@3DP-film, biphasic 3DP-film and ATV/PG-Lip@3DP-film, respectively. These results could be interpreted considering percentage swelling for films (Swelling, disintegration and erosion), where the fluid uptake by ATV/PG-Lip@3DP-film was lower than that for ATV@3DP-film, probably contributing to the more controlled drug release from the former.

The intermediate drug release rate from the biphasic 3DP-film, compared to ATV@3DP-film and ATV/PG-Lip@3DP-film, is expectedly related to the cumulative ATV release at different rates from both ATV@ink and ATV/PG-Lip@ink constituting the biphasic 3DP-film structure. These results further establish 3D printing as reliable customization tool for tailoring drug release profiles.

As detailed in Table 1S, drug release from ATV@3DP-film followed first order kinetics. Whereas kinetic analysis of the release profiles indicated that the best-fit model for both ATV/PG-Lip@3DP and biphasic film was the Higuchi diffusion model, which was consistent with various studies of bucco-adhesive films [51]. Korsmeyer-Peppas n values (Table 1S) supported diffusion-driven release.

### Mucoadhesive properties

Mucoadhesive films represent an efficient buccal pharmaceutical form; they provide retention for a longer period of time. In our work, we developed composite mucoadhesive 3DP-films, utilizing both HPMC and chitosan as mucoadhesive polymers [52]. Chitosan possesses mucoadhesive potential through the electrostatic interaction between its cationic amine groups and negatively charged mucin molecule [53]. However, it has some restrictions, which are mainly because of its low solubility and the weak mechanical strength of the formed gels. The mechanical strength of chitosan can be enhanced by blending with other polymers such as HPMC [54]. HPMC mucoadhesiveness is attributed to its non-ionic hydrophilicity leading to the diffusion and formation of interpenetration layer with mucus [53].

As shown in Fig. 5e, all the developed 3DP-films achieved an average mucoadhesive force of (2388.4 ± 18.4 dyne /cm^2^) and adhesiveness of (0.82 ± 0.025 mJ). Furthermore, mucoadhesion residence time for all the prepared films was beyond 24 h, indicating that inclusion of liposomal formulations within the polymer matrix did not affect the mucoadhesive attributes for liposomal films (PG-Lip@3DP-film and ATV/PG-Lip@3DP-film).

Taken together, the developed composite 3DP mucoadhesive films could afford reasonable swelling, acceptably controlled release and efficient mucoadhesive features. Thus, they would present good candidates for controlled buccal drug delivery.

### In vitro antifungal activity

In this work, we investigated the effect of the developed formulations on the reported ATV in vitro antifungal activity.

#### Determination of minimum inhibitory concentration (MIC)

The MIC of ATV solution and ATV/PG-Lip against four different fluconazole-resistant candida strains (two standard and two clinical isolates) was determined using agar dilution technique.

As can be seen in Table 3, both ATV solution and ATV/PG-Lip demonstrated antifungal activity against all tested strains. The determined MIC for ATV solution against *C. albicans* ATCC 10231 (32 µg/mL) agreed with previous data [10], while the MIC for ATV/PG-Lip was 128 µg/mL. The determined MIC against both *C. albicans* and *C. parapsilosis* clinical isolates for ATV solution and ATV/PG-Lip were 64 and 256 µg/mL, respectively.

**Table 3:** MIC of ATV solution and ATV/PG-Lip against different Candida strains

**Strain**^a^	**MIC (µg/mL)**	
	**ATV solution**	**ATV/PG-Lip**
*C. albicans *ATCC 10231	32	128
*C. albicans *ATCC 231GI	16	64
*C. albicans* (clinical isolate)	64	256
*C. parapsilosis* (clinical isolate)	64	256

^a^All-tested strains are fluconazole resistant

The higher MIC for ATV/PG-Lip compared to ATV solution can be attributed to the confinement of ATV in the liposomal vesicle (ATV/PG-Lip) which is further immobilized within the agar gel matrix, as imposed by the experimental setting. In addition, the lower diffusion of liposomal vesicles compared to ATV solution through the agar gel might have resulted in the lower overall interaction between drug in ATV/PG-Lip and candida cells. This result highlights the challenges facing microbiological testing of nanoparticle dispersions. It also confirms the need for other elaborate testing adopting different microbiological techniques, as performed in our study.

#### Time-dependent antifungal activity 

The growth curve for *C. albicans* ATCC 10231 was plotted to monitor the antifungal activity of ATV and ATV/PG-Lip (at MIC of 32 and 128 µg/mL, respectively) over time.

**Fig. 6:**
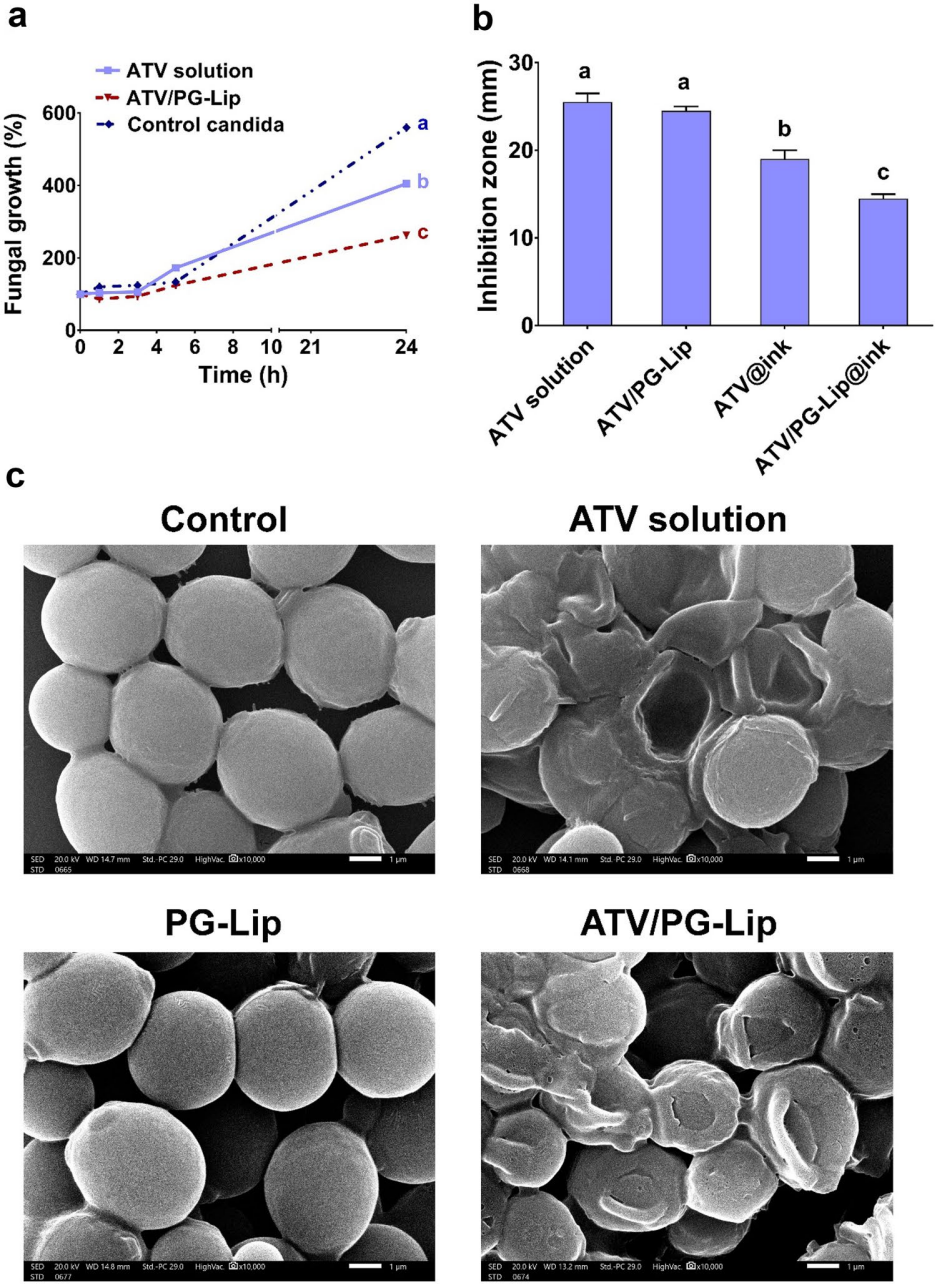
In vitro antifungal activity (**a**–**c**). Antifungal activity time profile for MIC values of ATV (32 µg/mL) and ATV/PG-Lip (128 µg/mL) (**a**). Fungal inhibition zones of different formulations (**b**). Data indicates mean ± SD, n = 3, different letters indicate statistically significant difference: a > b > c, at p ≤ 0.05. SEM micrographs of control *C. albicans* ATCC 10231 cells and cells treated for 2 h with MIC values of ATV solution (32 µg/mL) and ATV/PG-Lip (128 µg/mL) in comparison to PG-Lip (**c**). Micrographs are taken at (× 10,000), scale bars = 1 µm

Results clearly showed that percentages fungal growth was in order of control untreated candida > ATV solution > ATV/PG-Lip. Moreover, the fungal growth of ATV/PG-Lip (262.3 ± 4%) was significantly (p ≤ 0.05) lower than ATV solution (405 ± 5%) after 24 h (Fig. 6a). This finding may be due to the high solubilization of ATV as ATV/PG-Lip and its subsequent sustained release. Also, this may be attributed to the ability of vesicles to bind to fungal cell wall, facilitating the drug penetration to fungal cells [55].

#### Agar diffusion assay

The antifungal activity of different formulations was investigated against *C. albicans* 10231 strain using agar diffusion technique for 24 h. ATV solution and ATV/PG-Lip were tested in comparison to ATV@ink and ATV/PG-Lip@ink (all containing 250 µg ATV). Also, PG-Lip and plain polymer ink have been included. For normalization of ATV solution in DMSO inhibition zone, the inhibition zone of control DMSO was subtracted.

As can be clearly seen in Figs. 6b and 3S, the inhibition zone for ATV (25.5 ± 1 mm) was not significantly (p > 0.05) different from that for ATV/PG-Lip (24.5 ± 0.5 mm), which may be attributed to the ATV release from ATV/PG-Lip. The same finding was previously reported [56], where both free and liposomal cefepime exhibited similar inhibition zones.

The fungal inhibition zone for ATV@ink (19 ± 1 mm) was significantly (p ≤ 0.05) higher than ATV/PG-Lip@ink (14.5 ± 0.5 mm; Fig. 6b). This may be related to ATV entrapment in the liposomes which are further contained within the ink polymer blend. Both PG-Lip and plain polymer ink demonstrated no fungal inhibition (Fig. 3S).

#### Scanning electron microscope study

The effect of ATV and ATV/PG-Lip (at MIC of 32 and 128 µg/mL, respectively) on the ultrastructural features of *C. albicans* 10231 was investigated using SEM. For comparison, PG-Lip was included in the study.

As clearly demonstrated in Fig. 6c, control *C. albicans* ATTC 10231 exhibited a smooth surface and typical round morphology. Whereas cells treated with both ATV solution and ATV/PG-Lip exhibited drastic morphological alterations with obvious cellular shrinkage and rupture, implying prominent antifungal activity. Interaction of liposomal vesicles with fungal cells has been previously reported [55] and could further justify the resulting higher antifungal activity of ATV/PG-Lip compared to ATV solution in this study. Fungal cells treated with PG-Lip (Fig. 6c) showed no effect on fungal cell integrity.

Comparing PG-Lip to ATV/PG-Lip verified the hypothesis that drug loaded liposomes enhanced antifungal activity compared to blank liposomes [57]. This corroborates the obtained results of the time-dependent antifungal activity (Time-dependent antifungal activity).

### In vivo antifungal activity 

#### ELISA of inflammatory biomarkers 

ELISA was performed for the quantification of inflammatory biomarkers for different tested groups.

**Fig. 7:**
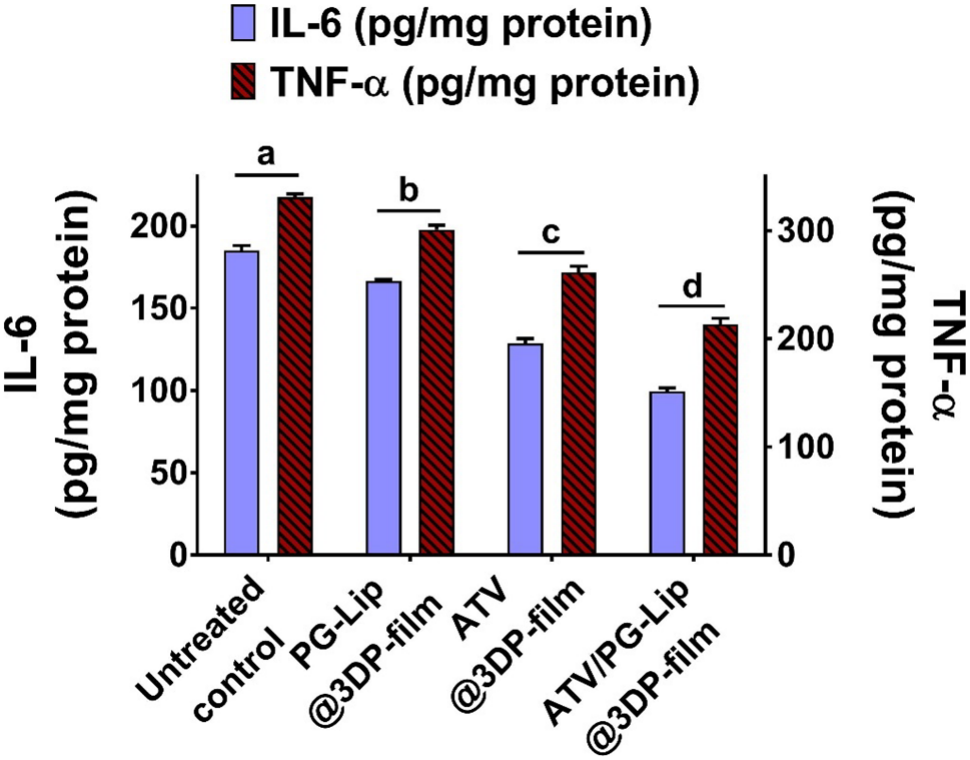
Level of pro-inflammatory cytokines TNF-α and IL-6 in tongue tissue for in vivo model. Data indicates mean ± SD, n = 3. Different letters indicate statistically significant difference: a > b > c > d at p ≤ 0.05

As shown in Fig. 7, infection-induced inflammation was evident for the untreated control group by the pronounced upregulation of both TNF-α (331 ± 3 pg/mg protein) and IL-6 (185 ± 3 pg/mg protein). Treatment significantly (p ≤ 0.05) reduced the pro-inflammatory cytokines compared to the untreated group. Notably, the amelioration of inflammation followed the pattern: ATV/PG-Lip@3DP-film > ATV@3DP-film > PG-Lip@3DP-film. Statins have been associated with an anti-inflammatory action against candidiasis-associated inflammation [10]. In our work, ATV/PG-Lip@3DP-film fostered superior anti-inflammatory potential. This goes in line with previous results, demonstrating the enhanced anti-inflammatory effect of simvastatin-loaded liposomes compared to simvastatin alone in vitro in foam cells [58].

#### Histological and histomorphometric analysis

**Fig. 8:**
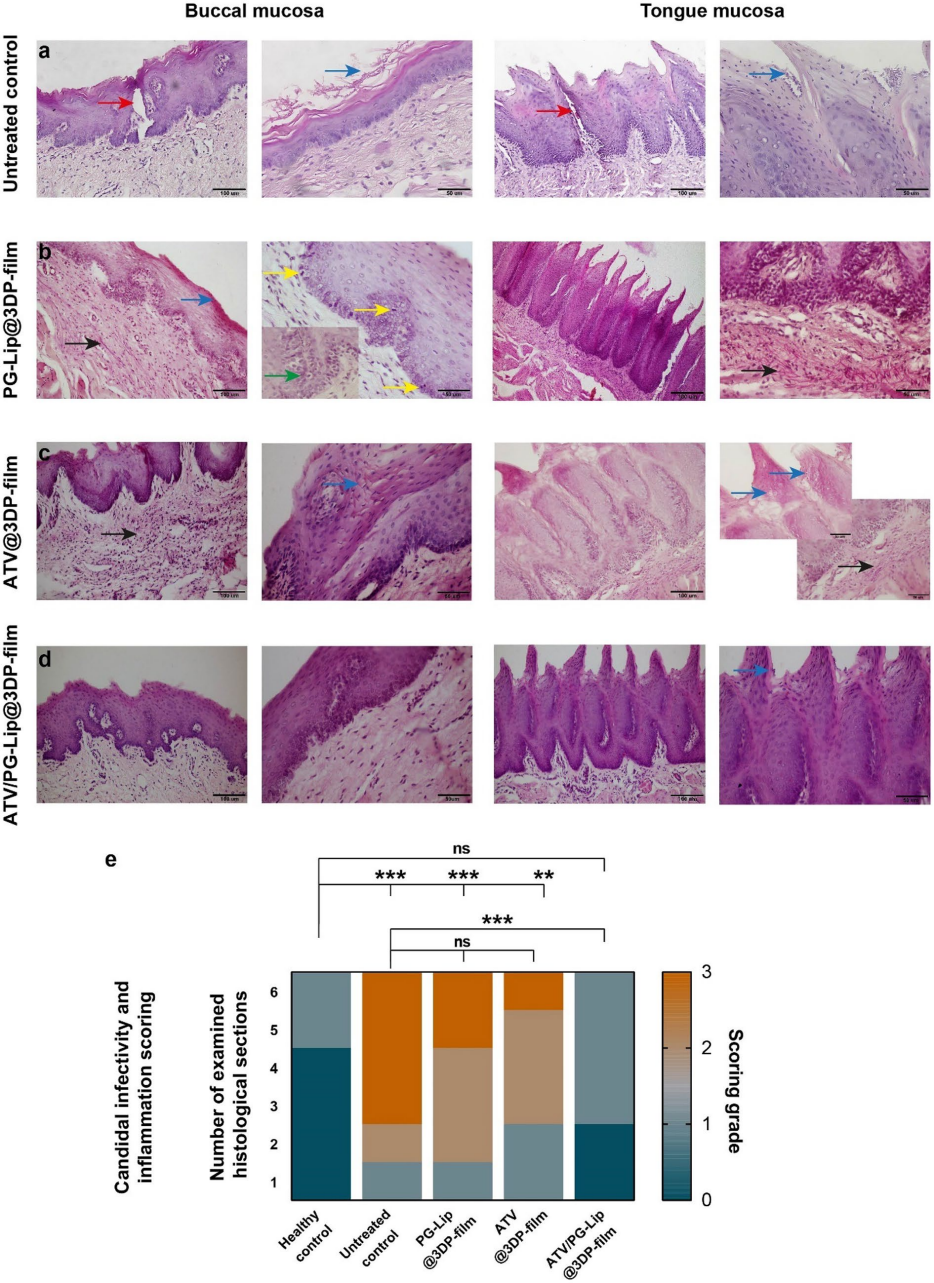
PAS-stained photomicrographs of the *C. albicans* infected rabbit buccal and tongue mucosa with/out treatment (**a**–**e**). The untreated mucosa displays the persistence of the hyphae and spores at the surface epithelium (blue arrows), inducing mild dysplastic changes in terms of pleomorphism, hyperchromatism and basilar hyperplasia in the basal epithelial third. Red arrows denote the candidal-induced intraepithelial macro abscesses (**a**). The PG-Lip@3DP-film group reveals cheek mucosa with acanthotic stratified squamous epithelium, showing signet ring fungal infected epithelial cells (green arrow in the inset). In both buccal and tongue mucosae, the PAS positive hyphae (blue arrows) occupy the upper 2/3 of the epithelium with evident compressed hyphae in the connective tissue (black arrows). Yellow arrows point out the mitotic activity induced by candidal infection (**b**). The buccal and lingual mucosae treated with ATV@3DP-film reveals candidal hyphae/spores within the epithelial (blue arrows) and subepithelial layers (black arrows), eliciting diffuse submucosal inflammation (**c**). Except for the sporadic spores (blue arrow) detected in the upper cornified layer of the lingual papillae, none of the candidal hyphae nor spores are present in the nonkeratinized cheek mucosa treated ATV/PG-Lip@3DP-film (**d**). Scale bar = 100 µm (× 200 lens magnification power) and = 50 µm (× 400 magnification). The morphometric heat-map for candida infectivity and inflammation score (**e**), revealing the antimycotic activity of the ATV/PG-Lip@3DP-film with restoring the score almost to the baseline healthy one. n = 6 measurements. The ** denotes p < 0.01, *** denotes p < 0.001, while non significance (ns) means p > 0.05

The in vivo model of oral candidiasis in rabbits has been previously developed [34]. In this work, it was further established, where the mucosa of the untreated control group demonstrated obvious signs of fungal infection (Fig. 8a). More specifically, compared to the mucosa of healthy control group (with 0 score, Fig. 3S), the buccal mucosa of untreated control group displayed candidal hyphae entangled with spores at the upper most layer of the epithelium, which itself became keratinized. Invasion of the hyphae and spores deep in the epidermal layer was evident, possibly inciting the acute immunoreaction identified as transepithelial neutrophil infiltration from the underlying stroma forming Munro’s abscess, with a significantly (p < 0.001) higher candida infectivity and inflammation score (score of 3) than healthy control group. The infected tongue mucosa for untreated group presented similar histopathological picture with noticeable candidal leukoplakic epithelial changes and scattered hyphae/spores within the lamina propria, provoking mild inflammatory cell reaction.

For PG-Lip@3DP-film group, a fair antifungal activity could be noted (Fig. 8b), where candidal hyphae were confined to the superficial epithelial layers (candida infectivity and inflammation score of 2). However, an initial defense mechanism was spotted as clear ballooning of epithelial cells in the basal third pointing out the intraepithelial inclusion of microorganism. Surprisingly, a condensation of candidal hyphae underneath the epithelial-connective tissue interface was identified (more obvious in the tongue lamina propria; Fig. 8b) probably a sign of immune sensitization. The noted antifungal activity for PG-Lip@3DP-film group could be possibly related to the antifungal activity of the employed matrix composition [59, 60].

On the other hand, the buccal mucosa and distant tongue mucosa for ATV@3DP-film group did not show pronounced antimycotic activity, scoring non significantly (p > 0.05) different score (score of 2) than untreated control group. In the buccal mucosa, the candida hyphae could be seen entangled within the upper half of the epithelium (Fig. 8c). Moreover, a diffuse stromal inflammatory reaction was evident, probably provoked by the escaped hyphae. These findings are in line with previous data [10], where the antifungal activity of fluvastatin solution was challenged both in vitro and in vivo in a mice model of intra-abdominal candidiasis. Although fluvastatin solution presented a pronounced antifungal activity in vitro (MIC = 8 µg/mL; against *C. albicans* ATCC 10231), it afforded no antifungal activity in vivo. Such behavior was assigned to the fact that statins may stimulate virulence factors that favor infection, hence increasing the fungal invasiveness. Moreover, the immunomodulatory effect of statins may help in weakening the response against candida [10].

Interestingly, ATV/PG-Lip@3DP-film fostered a prominent antifungal efficacy, securing the significantly (p < 0.001) lowest candida infectivity and inflammation score of 1, among treatment groups (Fig. 8d). Sporadic hyphae could be seen constrained within superficial layers of the buccal mucosa. Moreover, the antifungal activity was also evident for the tongue mucosa, almost restoring the normal architecture of the lingual submucosa with clearing of the invaded hyphae. In fact, ATV/PG-Lip@3DP-film group presented a non-significant (p > 0.05) difference in the candida infectivity and inflammation score (score of 1) from the healthy control group (score of 0; Fig. 8e). These findings imply the efficiency of ATV/PG-Lip as nanocarrier for boosting the antifungal efficacy of ATV, which further corroborates the superiority of the developed nanocarrier established by in vitro microbiological studies (In vitro antifungal activity). The formulated ATV/PG-Lip might have favorably contributed higher drug permeation/deposition into infected tissues, resulting in an overall higher antifungal functionality. This goes in line with enhanced antifungal efficacy of liposomal gel compared to plain fluconazole solution gel in the treatment of cutaneous candidiasis, as previously reported [61]. This was attributed to fluconazole sustained release from liposomal gel and its high localization in different strata of the skin compared to the plain gel [61].

## Conclusion

In the present work, we managed to address antifungal drug resistance via intertwining of drug repurposing, nanodrug delivery and 3D printing technique. The novel ATV/PG-Lip presented a favorable nanosize range with efficient ATV loading, in vitro cytocompatibility and three-month colloidal stability. Following the optimization of an innovative biofunctional ink containing chitosan, PVA and HPMC, we integrated ATV/PG-Lip into a mucoadhesive 3DP-film. The mucoadhesive 3DP films achieved controlled water uptake, disintegration and ATV release, while presenting acceptable mucoadhesive force. The formulated ATV/PG-Lip managed to prominently inhibit the growth of standard and clinically isolated fluconazole-resistant *C. albicans* strains. Finally, ATV/PG-Lip@3DP-film fostered a prominent antifungal efficacy and ameliorated inflammation in vivo in an oral candidiasis rabbit model. These findings establish the efficient employment of drug repurposing as a promising approach for tackling antimicrobial drug resistance. Moreover, the results highlight the role of nanodrug delivery as an impactful tool for therapeutic modification of drug efficacy. Finally, 3D printing technique remains a reliable customization tool for tailoring drug delivery devices. The developed mucoadhesive 3DP films can be envisioned as affordable and convenient antifungal patches for self-medication of oral candidiasis with high patient compliance.

### Supplementary Information

Below is the link to the electronic supplementary material.

Supplementary file1 (PDF 566 KB)

## Data Availability

The authors confirm that the data for this study findings are available within the article and the supplementary information file.

## Declarations

### Animal studies

All institutional and national guidelines for the care and use of laboratory animals were followed.
