# Supplementary material for: Atorvastatin liposomes in a 3D-printed polymer film: a repurposing approach for local treatment of oral candidiasis
Source: Drug Deliv Transl Res. 2023 May 15;13(11):2847–68. doi: 10.1007/s13346-023-01353-4 (PMC10545585; doi:10.1007/s13346-023-01353-4)
Supplement: Supplementary file 1 — Supplementary file1 (PDF 566 KB) [file 13346_2023_1353_MOESM1_ESM.pdf]

Atorvastatin liposomes in a 3D-printed polymer film: a repurposing approach for local treatment of oral candidiasis

Drug Delivery and Translational Research

Supplementary material

Eman M. Nour[a], Salma E. El-Habashy[a], Michael G. Shehat[b], Marwa M. Essawy[c, d], Riham M. El-Moslemany[a], Nawal M. Khalafallah[a]

[a] *Department of Pharmaceutics, Faculty of Pharmacy, Alexandria University, Alexandria, Egypt.*

[b] *Department of Microbiology and Immunology, Faculty of Pharmacy, Alexandria University, Alexandria, Egypt.*

[c] *Department of Oral Pathology, Faculty of Dentistry, Alexandria University, Alexandria, Egypt.*

[d] *Center of Excellence for Research in Regenerative Medicine and Applications (CERRMA), Faculty of Medicine, Alexandria University, Alexandria, Egypt.*

** **Corresponding author**: Salma E. El-Habashy, Department of Pharmaceutics, Faculty of Pharmacy, Alexandria University, Alexandria, Egypt.*

1 Khartoum Square, Azarita, Alexandria, Egypt. P.O. Box 21521, Alexandria, Egypt.

***Tel:** +2 0109 193 5945*

***Email**: salma.elhaleem@alexu.edu.eg, dr.salma.essam@hotmail.com*

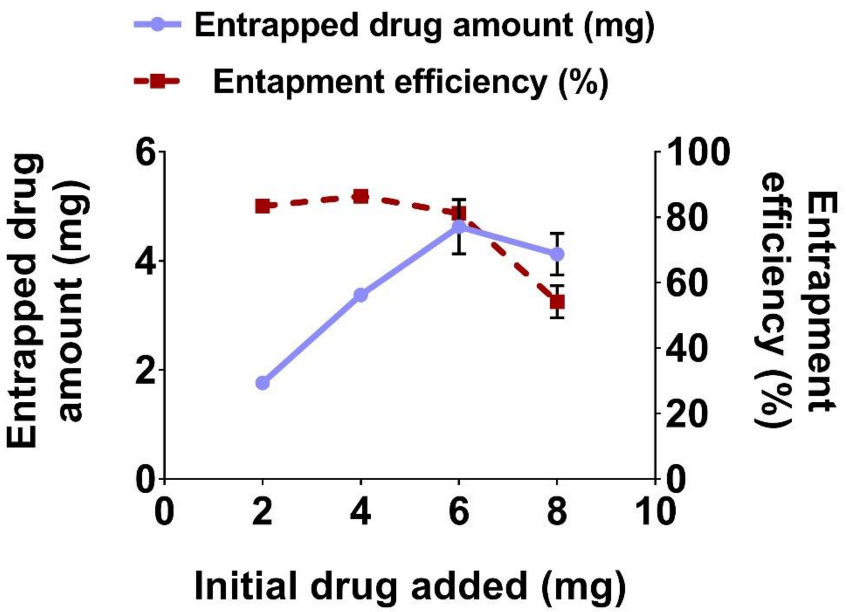

Fig. 1S Results of ATV entrapment in ATV/PG-Lip using different initial drug concentrations. *Data indicates mean ± SD, n = 3, p ≤ 0.05*

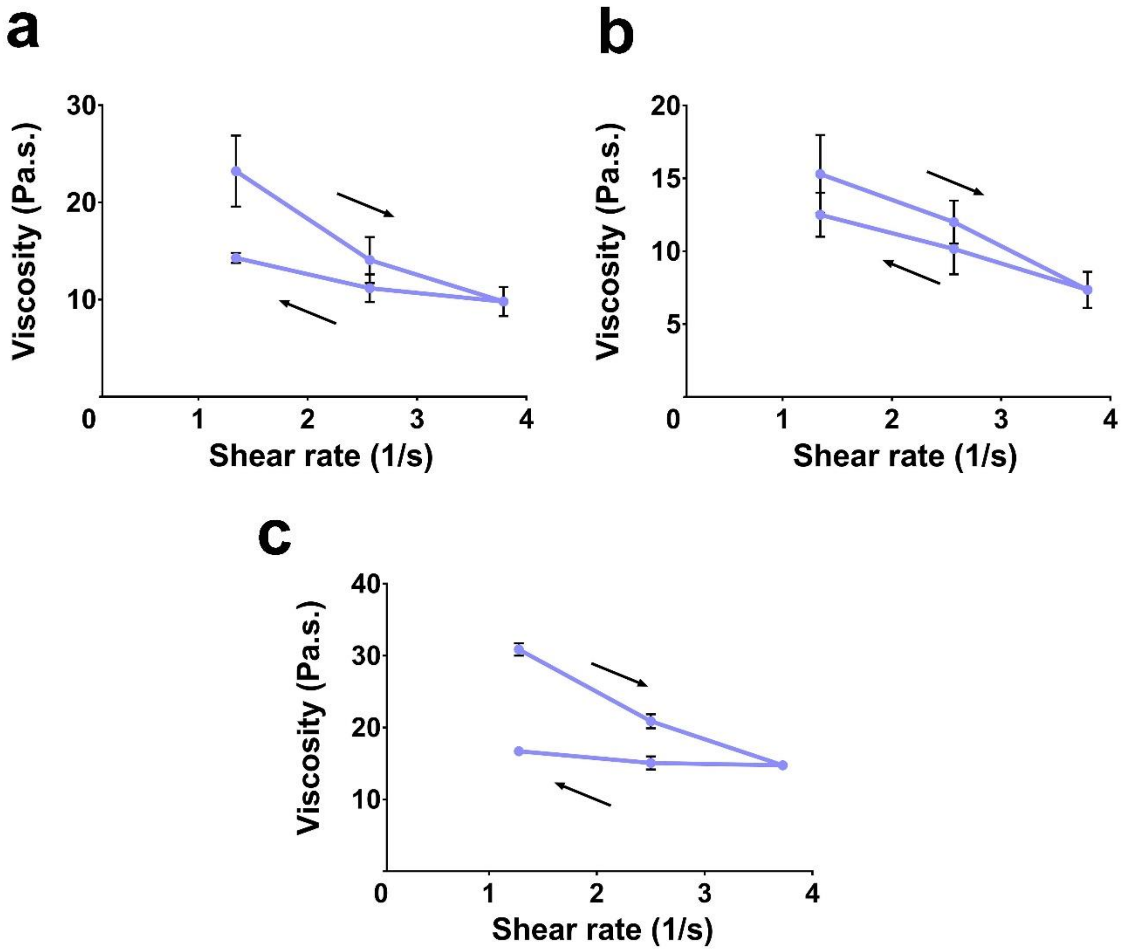

Fig. 2S Viscosity values of the prepared inks containing 3% (w/v) PVA, 3% (w/v) HPMC and 5% (w/v) chitosan at different shear rates (a-c). *Curves for ATV@ink* ***(a),*** *PG-Lip@ink* ***(b)*** *and ATV/PG-Lip@ink* ***(c)***

Table 1S: Drug release kinetics modeling for different ATV formulations

**Formulations**	**Release kinetics parameters** [a]				
	**Zero order**	**First order**	**Higuchi**	**Korsmeyer-Peppas**	
	**R^2**	**R^2**	**R^2**	**R^2**	**n**
ATV solution	0.99 ± 0.001	0.92 ± 0.003	0.83 ± 0.001	0.99 ± 0.001	0.92 ± 0.015
ATV/PG-Lip	0.13 ± 0.017	0.87 ± 0.022	0.97 ± 0.005	0.96 ± 0.008	0.44 ± 0.003
ATV@3DP-film	0.74 ± 0.064	0.98 ± 0.002	0.87 ± 0.014	0.85 ± 0.001	0.33 ± 0.005
ATV/PG-Lip@3DP-film	0.57 ± 0.076	0.79 ± 0.001	0.98 ± 0.007	0.93 ± 0.012	0.31 ± 0.006
Biphasic 3DP-film	0.56 ± 0.084	0.96 ± 0.001	0.99 ± 0.005	0.91 ± 0.008	0.31 ± 0.007

[a] Data indicates mean ± SD.

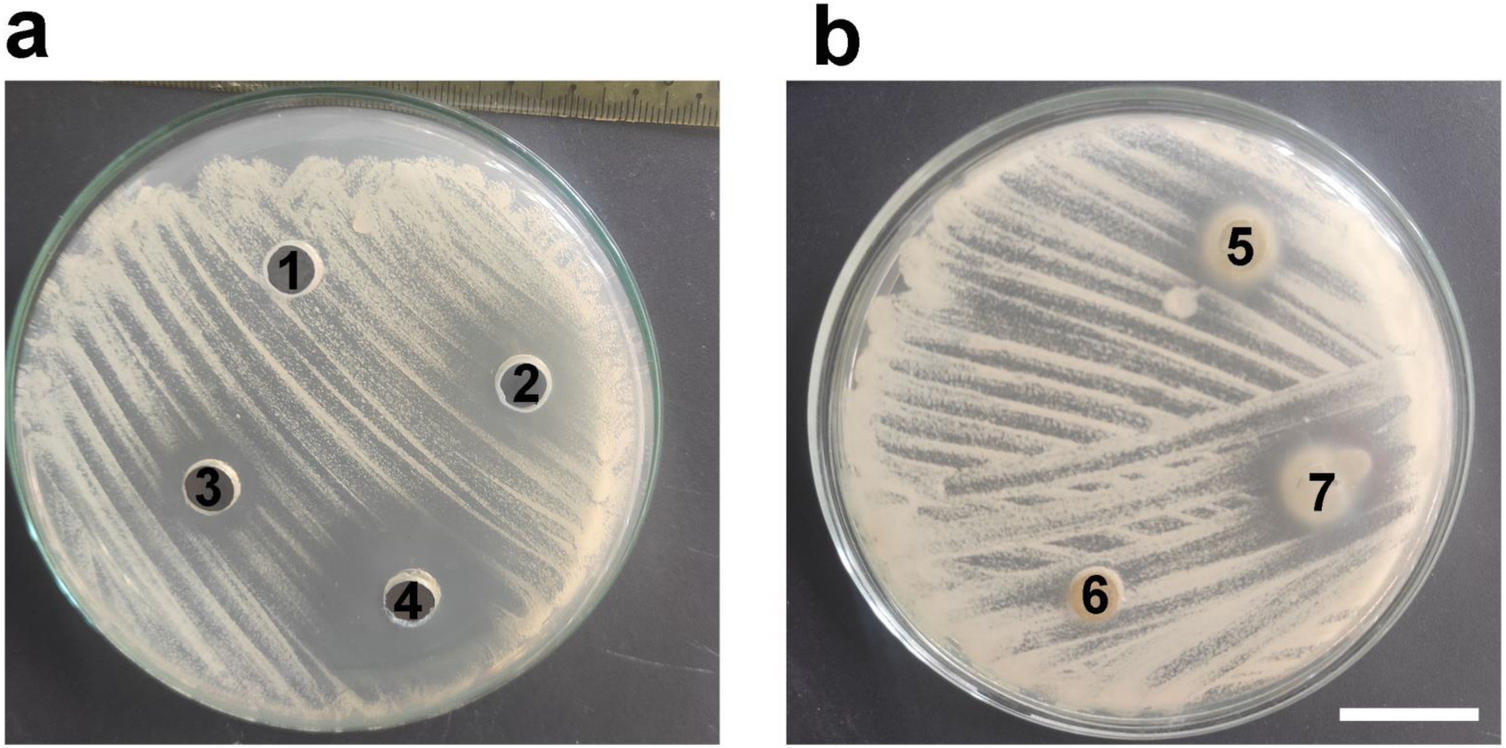

Fig. 3S Inhibition zones of different formulations on Muller-Hinton agar (a and b), *corresponding to 250 µg ATV. PG-Lip (1), ATV/PG-Lip (2), DMSO (as control) (3), ATV solution (4) on agar plate* ***(a).*** *ATV/PG-Lip@ink (5), Plain polymer ink (6), ATV@ink (7) on agar plate* ***(b).*** *Scale bar = 2 cm*

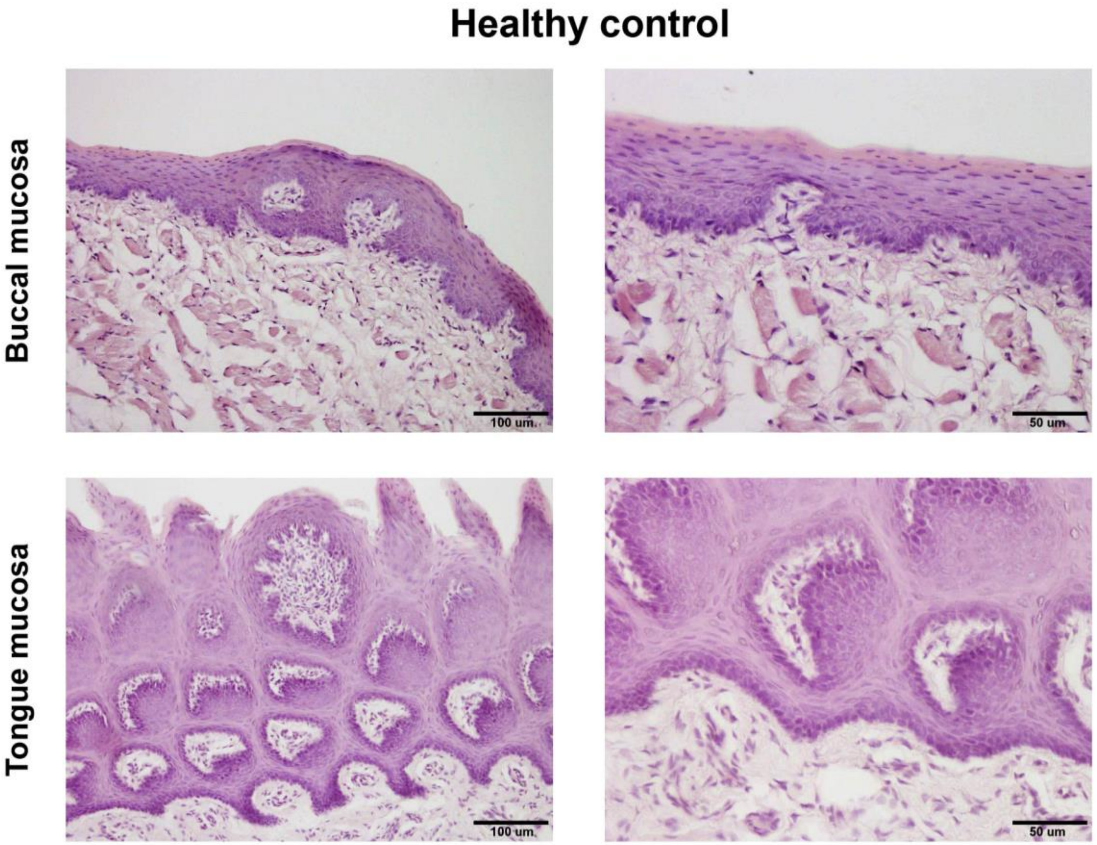

Fig. 4S *A photomicrograph of PAS-stained normal rabbit buccal and tongue mucosa revealing nonkeratinized and orthokeratinized stratified squamous epithelium, respectively, without inflammatory infiltrate in their loose lamina propria. Scale bars = 100 µm (×200 magnification) and 50 µm (×400 magnification)*
